# Differential expression analysis using a model-based gene clustering algorithm for RNA-seq data

**DOI:** 10.1186/s12859-021-04438-4

**Published:** 2021-10-20

**Authors:** Takayuki Osabe, Kentaro Shimizu, Koji Kadota

**Affiliations:** 1 Graduate School of Agricultural and Life Sciences, The University of Tokyo, Yayoi 1-1-1, Bunkyo-ku, Tokyo, 113-8657 Japan; 2 Collaborative Research Institute for Innovative Microbiology, The University of Tokyo, Yayoi 1-1-1, Bunkyo-ku, Tokyo, 113-8657 Japan; 3 Interfaculty Initiative in Information Studies, The University of Tokyo, Hongo 7-3-1, Bunkyo-ku, Tokyo, 113-0033 Japan

**Keywords:** RNA-seq, Differential expression, Gene clustering, Posterior probability

## Abstract

**Background:**

RNA-seq is a tool for measuring gene expression and is commonly used to identify differentially expressed genes (DEGs). Gene clustering is used to classify DEGs with similar expression patterns for the subsequent analyses of data from experiments such as time-courses or multi-group comparisons. However, gene clustering has rarely been used for analyzing simple two-group data or differential expression (DE). In this study, we report that a model-based clustering algorithm implemented in an R package, MBCluster.Seq, can also be used for DE analysis.

**Results:**

The input data originally used by MBCluster.Seq is DEGs, and the proposed method (called MBCdeg) uses all genes for the analysis. The method uses posterior probabilities of genes assigned to a cluster displaying non-DEG pattern for overall gene ranking. We compared the performance of MBCdeg with conventional R packages such as edgeR, DESeq2, and TCC that are specialized for DE analysis using simulated and real data. Our results showed that MBCdeg outperformed other methods when the proportion of DEG (*P*_*DEG*_) was less than 50%. However, the DEG identification using MBCdeg was less consistent than with conventional methods. We compared the effects of different normalization algorithms using MBCdeg, and performed an analysis using MBCdeg in combination with a robust normalization algorithm (called DEGES) that was not implemented in MBCluster.Seq. The new analysis method showed greater stability than using the original MBCdeg with the default normalization algorithm.

**Conclusions:**

MBCdeg with DEGES normalization can be used in the identification of DEGs when the *P*_*DEG*_ is relatively low. As the method is based on gene clustering, the DE result includes information on which expression pattern the gene belongs to. The new method may be useful for the analysis of time-course and multi-group data, where the classification of expression patterns is often required.

**Supplementary Information:**

The online version contains supplementary material available at 10.1186/s12859-021-04438-4.

## Background

RNA-seq is commonly used to obtain genome-wide expression data for genes [1, 2] and to identify differentially expressed genes (DEGs) for different groups or conditions [3, 4]. To date, several methods to enable the analysis of RNA-seq data have been developed, including normalization [5–10], various R packages [11–16], and graphical user interfaces (GUI) [17–19]. Research on more efficient and accurate methods to identify DEGs continues, and new findings continue to be reported by researchers [20–25].

Obtaining a ranked gene list for the degree of differential expression (DE) is a starting point for gaining biological insights into the groups being compared [26]. Several analysis methods such as gene ontology and the construction of co-expression networks have been used to examine the biological mechanisms underlying DEGs [25]. Additionally, gene clustering based on the similarity of expression patterns has been widely used to group and classify DEGs [27–29]. Gene clustering has also been used for time-course and multi-group data in which several expression patterns may exist. However, the method is usually not used to classify DEGs obtained by two-group comparisons which is the minimum size for a comparative analysis and is used even less to identify DEGs.

In this study, we propose the use of model-based clustering algorithms for the identification of DEGs from RNA-seq count data. Although various sophisticated algorithms for gene clustering are available [30–32], we focus our analysis on the R package MBCluster.Seq [28], as its framework is compatible with DE analysis and it is comparable with other R packages dedicated to detecting DEGs. We describe the application of the model-based gene clustering method and the incorporation of a robust normalization algorithm called DEGES [7, 14] for detecting DEGs. Next, we compare the method with other competing packages dedicated to DE analysis such as edgeR [11], DESeq2 [15], and TCC [14] using simulated data and real data. Further, we discuss the potential limitations of the method and present a set of guidelines for practical use.

## Results

### Proposed method for identification of DEGs based on gene clustering

In this study, we devised a new analysis method that uses the functionalities of the MBCluster.Seq package. Therefore, for consistency and clarity, we used notations that were similar to those described in the original paper [28]. First, we outlined a method to describe the input/output relationship. We denote an input count matrix as one where each row indicates a gene *g* (= 1, …, *G*), each column a replicate *j* (= 1, …, *n*_*i*_) of group *i* (= 1, …, *I*), and each cell the number of counts. Here, *G* is the number of genes, *I* is the number of compared groups, and *n*_*i*_ is the number of replicates for the group *i*. MBCluster.Seq clusters gene vectors *β*_*g*_ = (*β*_*g*1_, …, *β*_*gI*_), where *β*_*gi*_ indicates the count of gene *g* in the group *i* relative to the overall mean on a log-scale. Therefore, the summation of *β*_*gi*_ for a gene *g* across all compared groups was 0. *β*_*g*_ can be described as the log fold-change (*FC*) between compared groups. Given a preselected number of clusters *K*, MBCluster.Seq gives two results as an output. One is the center for cluster *k*, *μ*_*k*_ = (*μ*_*k*1_, …, *μ*_*kI*_) for *k* = 1, …, *K*, and the other is the posterior probability (PP) that gene *g* belongs to the *k*th cluster, *p*_*g*_ = (*p*_*g*1_, …, *p*_*gK*_) for *g* = 1, …, *G*.

A ranked gene list for a cluster mainly consisting of non-DEGs can be obtained via MBCluster.Seq by using the assigned PPs. To identify the non-DEG cluster, we consider the *L*^*2*^ Norm of *μ*_*k*_ for each cluster center across groups, ||*μ*_*k*_||_2_ = (|*μ*_*k*1_|^2^ + ⋯ +|*μ*_*kI*_|^2^)^1/2^. A smaller value of the norm for cluster *k* indicates a smaller degree of DE across groups for the cluster. Accordingly, we can regard the probability of a gene being located in the *k*th column as that in the non-DEG cluster, where *k* = argmin(||*μ*_1_||_2_, …, ||*μ*_*K*_||_2_). A lower value of the PP for gene *g* in the *k*th cluster (i.e., *p*_*gk*_) indicates a higher degree of DE between the compared groups. For simplicity, we call the proposed method based on MBCluster.Seq as “MBCdeg”.

MBCluster.Seq employs a scaling normalization algorithm [33] as the default, where the upper quartile (i.e., 75th percentile) counts for individual samples are used as the scaling factors to obtain equal values for upper quartile counts across all samples after the normalization. This type of conventional normalization algorithm assumes that the proportion of DEGs (*P*_*DEG*_) in the data is small (e.g., less than 5%) or that the proportion of genes upregulated in individual groups in the DEGs is approximately balanced (i.e., *P*_1_ ≈ *P*_2_ when comparing groups 1 and 2; *P*_1_ + *P*_2_ = 1) [34–36]. However, in practice, these assumptions are invalid in certain cases, such as when *P*_*DEG*_ ≈ 60% [37] and *P*_1_ >  > *P*_2_ [38]. To investigate the effect of various normalization algorithms on the data analysis, we employed a competing method (called DEGES) [7, 14]. The main arguments in its favor are (1) DEGES was originally designed to manipulate such scenarios (~ 25% of *P*_*DEG*_ with *P*_1_ >  > *P*_2_), (2) it can be applied to ~ 60% of *P*_*DEG*_ [35], and (3) the output (i.e., normalization factors) can easily be applied to the framework of MBCluster.Seq. Henceforth, we refer to MBCdeg using the default normalization algorithm and with the DEGES normalization algorithm as “MBCdeg1” and “MBCdeg2”, respectively.

### Analysis of simulated data for a two-group comparison

We show an example of MBCdeg based analysis for simulated data for two groups: *G* = 10,000, *n*_1_ = *n*_2_ = 3, *P*_*DEG*_ = 0.25, *P*_1_ = 0.9 (or *P*_2_ = 0.1), and *FC* = 4. We performed a run for MBCdeg with *K* = 3, assuming three expression patterns: up-regulated in group 1 (“DEG1” pattern), up-regulated in group 2 (“DEG2”), and consistent in both groups (“non-DEG”). Using this approach, we ideally obtain the centers for the cluster *k* (= 1, 2, 3) as *μ*_1_ = (0.69, − 0.69), *μ*_2_ = (− 0.69, 0.69), and *μ*_3_ = (0.00, 0.00), with log_e_(2) = 0.69. Clearly, the third cluster has the smallest *L*^2^ Norm (i.e., ||*μ*_3_||_2_ = 0). We therefore regard this cluster as containing many non-DEGs and used the PPs assigned to this cluster for estimating the overall degree of DE.

Here, MBCdeg1 gives the output *μ*_1_ = (0.56, − 0.56), *μ*_2_ = (− 0.14, 0.14), and *μ*_3_ = (− 0.81, 0.81) and uses the PPs of the second cluster displaying the smallest norm (||*μ*_2_||_2_ = 0.195) for gene ranking. Meanwhile, MBCdeg2 outputs *μ*_1_ = (0.68, − 0.68), *μ*_2_ = (− 0.69, 0.69), and *μ*_3_ = (− 0.02, 0.02) and uses the PPs of the third cluster displaying the smallest norm (||*μ*_3_||_2_ = 0.027) for gene ranking. The expression patterns for the cluster centers from MBCdeg2 were closer to the ideal patterns than those from MBCdeg1. This result suggests that the DEGES normalization may be useful in the framework for MBCdeg, at least in similar scenarios.

Next, we evaluated the performance of the overall gene ranking using the area under the receiver operating characteristic (ROC) curve (i.e., AUC). The AUC enables data comparisons without a tradeoff in sensitivity and specificity because the ROC curve is created by plotting the true positive rate (i.e., sensitivity) against the false positive rate (1 − specificity) obtained for each possible threshold value [39–41]. Therefore, an accurate method should provide high AUC values. We compared five methods: two clustering-based methods (MBCdeg1 and MBCdeg2) and three conventional methods (edgeR [11], DESeq2 [15], and TCC [14]). The edgeR and DESeq2 packages are widely used [25]. TCC is used as the main alternative to MBCdeg as it utilizes the DEGES normalization algorithm.

**Fig. 1:**
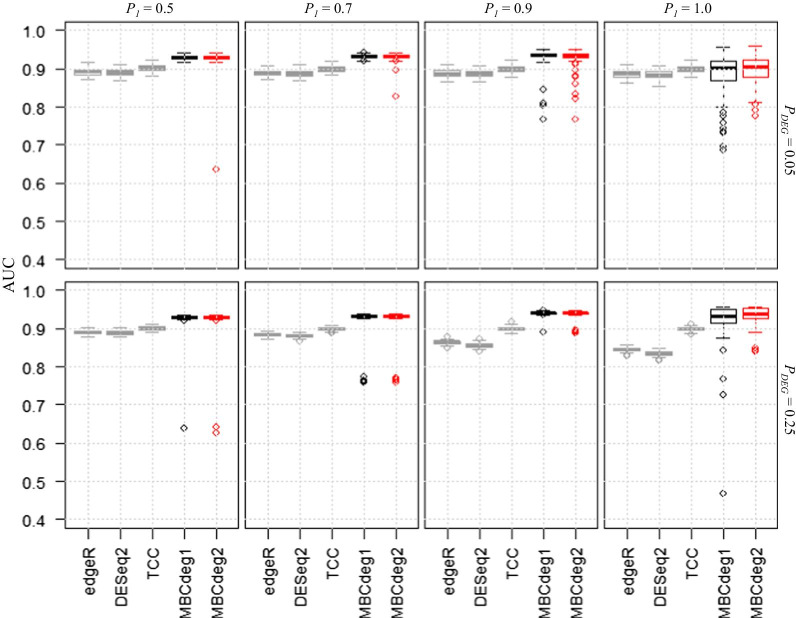
Results for two-group simulated data (*P*_*DEG*_ ≤ 0.25). Boxplots of AUC values (100 trials) for five methods for a total of eight conditions, *P*_1_ = 0.5 (left) to 1.0 (right) with *P*_*DEG*_ = 0.05 (upper) and 0.25 (lower). The performance of MBCdeg (with *K* = 3) was high in most trials. The explanation for a representative trial (AUC = 0.9295) and the worst trial (AUC = 0.633) using MBCdeg2 with *P*_*DEG*_ = 0.25 and *P*_1_ = 0.5 is given in Table 1

Figure 1 shows the AUC values for the five methods using various simulations: *P*_*DEG*_ = 0.05 and 0.25, with *P*_1_ = 0.5, 0.7, 0.9, and 1.0. A higher *P*_1_ value indicates a higher degree of upregulated DEGs in group 1, ranging from symmetric (*P*_1_ = 0.5) to completely asymmetric (*P*_1_ = 1.0) conditions. The AUC values for the two MBCdeg methods were higher than those from the conventional DE methods. TCC showed the best among the three conventional methods because the simulated data was generated using this package and the DEGES normalization algorithm in TCC was originally designed to manipulate such asymmetric scenarios (i.e., *P*_1_ > 0.5). Therefore, the high performance of MBCdeg over TCC is an interesting result.

The performance of MBCdeg does not appear to depend on whether the simulation scenario is symmetric or asymmetric. Most of the trials of MBCdeg showed AUC values of 0.93, and the range of *P*_1_ from 0.5 to 0.9, suggesting that the framework of MBCdeg is intrinsically robust for both symmetric and asymmetric data. As indicated above, we confirmed that MBCdeg2 tends to have lower norm values for non-DEG clusters (i.e., ||*μ*_*k*_||_2_ with *k* = argmin(||*μ*_1_||_2_, …, ||*μ*_*K*_||_2_)) than MBCdeg1 in scenarios with *P*_1_ > 0.5. This can be explained by the use of DEGES in the MBCdeg2 algorithm.

The overall performance of MBCdeg2 was very similar to that of MBCdeg1 although the former performs better than the latter in completely asymmetric scenarios (i.e., *P*_1_ = 1.0). We hypothesized that the key to accurate gene ranking may be to identify non-DEG cluster, and not to make the ideal non-DE pattern. This trend was also observed when the number of replicates per group was increased to *n*_1_ = *n*_2_ = 6, 9, and 12 (Additional file 1). In a comparison of the performance of each method using different numbers of replicates (Fig. 1 and Additional file 1), we observed that AUC values tend to increase as the number of replicates increases and this trend is consistent with a previous report [20].

### Effect on different numbers of clusters

We noted that MBCdeg tends to increase the variation in AUC between trials in association with an increase in *P*_1_ values, and this effect was greatest at *P*_1_ = 1.0. For example, the AUC values for MBCdeg1 and MBCdeg2 were less than 0.8 for ten and three trials, respectively, when *P*_*DEG*_ = 0.05 (Fig. 1). The relatively poor performance of MBCdeg at *P*_1_ = 1.0 than with other values of *P*_1_ can be explained by the difference between the true number of clusters (i.e., *K*_*truth*_ = 2) in this condition and the number of clusters that were used (*K* = 3). The simulated data obtained with *P*_1_ = 1.0 has two expression patterns; “DEG1” and “non-DEG”, and does not have the “DEG2” pattern.

**Fig. 2:**
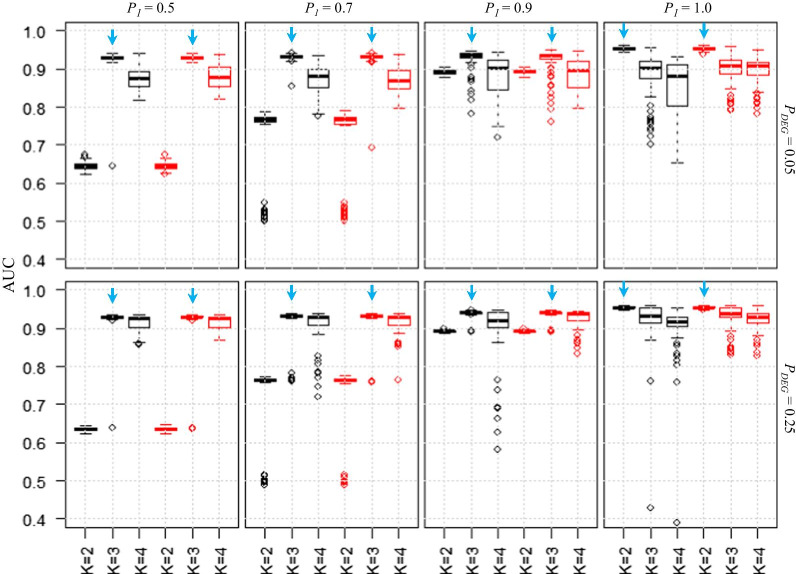
Effect of the different cluster numbers for MBCdeg (*K* = 2–4). Boxplots of AUC values (100 trials) for MBCdeg1 (*K* = 2–4; colored in black) and MBCdeg2 (*K* = 2–4; colored in red) are shown. The remaining legends are the same as in Fig. 1. The AUC values for MBCdeg with *K* = 3 were similar to those in Fig. 1 (different trials were used). Note that two trials result in AUC < 0.4 (not shown): MBCdeg1 with *K* = 2 with *P*_*DEG*_ = 0.05 and *P*_1_ = 0.9 (AUC = 0.3498), and MBCdeg1 with *K* = 4 with *P*_*DEG*_ = 0.25 and *P*_1_ = 1.0 (AUC = 0.3893)

To demonstrate the effect of using MBCdeg on a predefined number of clusters, we investigated the AUC values for MBCdeg with *K* = 2–4 (Fig. 2). Our results showed that the AUC values were the highest when *K* = *K*_*truth*_. The highest AUC values for *P*_1_ = 0.5, 0.7, 0.9, and 1.0 were obtained using *K* = 3, 3, 3, and 2, respectively (indicated by the downward arrow in light blue). This result suggests that the use of an accurate number of clusters is important when performing an analysis using MBCdeg. The distributions of the AUC values indicate the *K* values that should be used in practice. At *P*_1_ = 0.5 and 0.7 (i.e., *K*_*truth*_ = 3), the results for *K* > *K*_*truth*_ (i.e., *K* = 4) were more accurate than those with *K* < *K*_*truth*_ (i.e., *K* = 2). Further, this trend was observed when the number of replicates per group was increased to *n*_1_ = *n*_2_ = 6, 9, and 12 (Additional file 2).

In practice, it may be safe to adopt a larger *K* value. For two-group comparisons, the user would only need to run MBCdeg with *K* ≥ 3. Although the frequency of trials that result in a low performance of gene ranking is relatively high at larger *P*_1_ values, the probability of obtaining extremely asymmetric results such as *P*_1_ = 1.0 is low, and may at most indicate *P*_1_ = 0.9. Therefore, both methods (MBCdeg1 and MBCdeg2) may be useful in scenarios that are similar to the conditions investigated.

Although the probability of extremely low performance at *P*_1_ ≤ 0.9 is at most 2%, we should discuss the reasons. For example, the use of MBCdeg2 with *K* = 3 outputs one such result (AUC = 0.634) with *P*_*DEG*_ = 0.25 and *P*_1_ = 0.5. As this simulated data consists of 1250 DEG1, 1250 DEG2, and 7500 non-DEG patterns, almost all trials with high AUC values (> 0.92) gave three clusters, each of which consisted of many genes that resembled one of the three patterns, and having one predominant pattern (Table 1a). However, for the trial with low AUC value (0.634), the numbers of genes in the three clusters were 1299, 8670, and 31, respectively (Table 1b). Of these, the first cluster consisted of the most genes showing the DEG2 pattern, i.e., 12 DEG1, 935 DEG2, and 352 non-DEG patterns. The second cluster consisted of a great majority of genes with non-DEG patterns, i.e., 1238 DEG1, 289 DEG2, and 7143 non-DEG patterns. As this non-DEG cluster contains 1238/1250 = 99.0% of the genes with a true DEG1 pattern, these ranking inaccuracies may be responsible for the low AUC values obtained. As per our analysis, most low AUC values result due to the incorporation of DEGs (i.e., the DEG1 or DEG2 patterns) into the non-DEG clusters.

**Table 1:** Results for trials using MBCdeg2 with *P*_*DEG*_ = 0.25 and *P*_1_ = 0.5

	DEG1	DEG2	non-DEG	Total	*L*^2^ Norm
(a) AUC = 0.9295					
First cluster	884	3	317	1204	0.9792
Second cluster	3	900	222	1125	0.9779
Third cluster	363	347	6961	7671	0.0029
(b) AUC = 0.6336					
First cluster	12	935	352	1299	0.9546
Second cluster	1238	289	7143	8670	0.1904
Third cluster	0	26	5	31	1.7785
Truth	1250	1250	7500	10,000	

Two trial results with (a) high and (b) low AUC values as shown in Fig. 1 are described. MBCdeg2 was performed using *K* = 3, both trials output three clusters. The third and second clusters for the trials (a) and (b) were considered to be non-DEG clusters, respectively. For details, see the main text

The third cluster shown in Table 1b had very few genes (i.e., 26 DEG2 and 5 non-DEGs). These results indicate that the first two clusters determine the performance of the trial. The results from using *K* = 2 with the same simulation conditions (i.e., *P*_*DEG*_ = 0.25 and *P*_1_ = 0.5 in Fig. 2) indicate that the distribution of AUC values located around 0.63 may thus be reasonable. Similar inferences can be made for other results, for example, with *P*_*DEG*_ = 0.25 and *P*_1_ = 0.7. As the simulated data consists of 1750 DEG1, 750 DEG2, and 7500 non-DEG patterns, MBCdeg with *K* = 2 tends to output one cluster that is mainly composed of non-DEG and DEG2 genes, with the second cluster mainly composed of DEG1 genes. The median AUC values (≈ 0.77) when using MBCdeg with *K* = 2 at *P*_1_ = 0.7 are higher than those (≈ 0.63) at *P*_1_ = 0.5, and this can be explained by the smaller number of true DEG patterns (750 < 1250) contained in the non-DEG cluster. As this phenomenon was observed using both MBCdeg1 and MBCdeg2, it does not result due to the differences in the normalization algorithms. MBCdeg determines clusters stochastically, and therefore an output of results that appear as outliers will be obtained with a certain probability.

### Effect on different degrees of DE

For the simulation analysis described above, the degree of DE was fixed at fourfold (i.e., *FC* = 4), which limits the expression pattern of genes upregulated in a group to one, which is favorable when using MBCdeg. However, in practice, the degree of DE would differ for genes. Therefore, we performed simulations using different degrees of DE [7], where the *FC*s for DEGs were randomly sampled from “1.2 + a gamma distribution with shape = 2.0 and scale = 0.5.” Accordingly, the minimum and mean fold-change were approximately 1.2 and 2.2 (= 1.2 + 2.0 × 0.5), respectively.

The results of the analysis indicated that greater variability in the performance of MBCdeg is observed compared to the results obtained using fixed *FC*, as shown in Fig. 1 (Additional file 3). AUC values are lower overall than those from the fixed *FC* comparison (Fig. 1) in the same condition, and this can be explained by the lower degree of DE (fourfold → 2.2-fold). Though MBCdeg gave superior results than other packages (edgeR, DESeq2, and TCC) with *P*_*DEG*_ = 0.25, it did not perform as well using *P*_*DEG*_ = 0.05. This may occur due to the small number of DEGs (10,000 × 0.05 = 500 genes) under the conditions used for analysis and the resulting instability of clusters that mainly consisted of DEG1 or DEG2 patterns. As the total number of DEGs is relatively large with *P*_*DEG*_ = 0.25, clusters containing more DEG1 or DEG2 patterns are more likely to result and are probably more stable.

Notably, the AUC values for MBCdeg are not always the highest when *K* = *K*_*truth*_ (Additional file 4). With *P*_*DEG*_ = 0.25 and *P*_1_ = 0.9, the AUC values tend to increase as the number of clusters *K* increases. This may occur because the simulated data contains more expression patterns than *K*_*truth*_. In the conditions used here, *K*_*truth*_ = 3 was used for convenience. However, genes upregulated in a group may show various degrees of DE, such as 2.1-fold and 2.7-fold, and allowing the formation of separate clusters would have reduced the possibility of the inclusion of these genes in the non-DEG cluster.

### Effect on larger P_DEG_ values

**Fig. 3:**
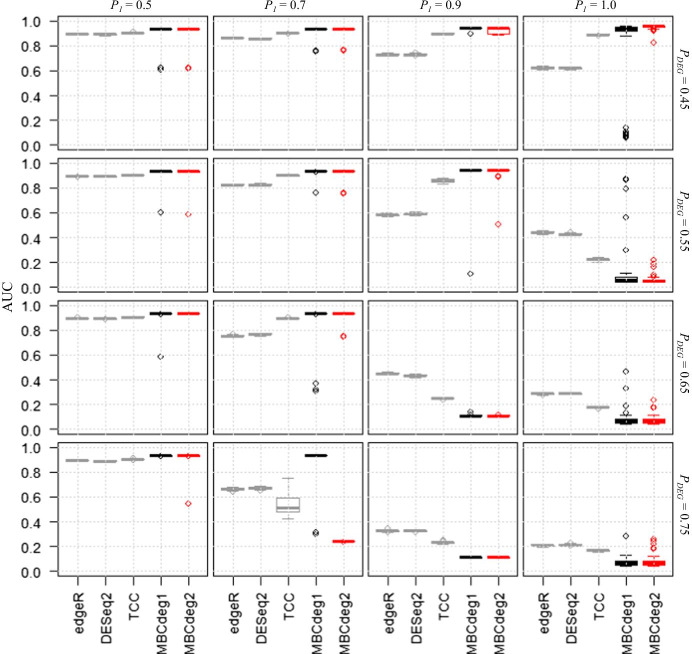
Effect on larger *P*_*DEG*_ values (*P*_*DEG*_ ≥ 0.45). Boxplots of AUC values (50 trials) for five methods for a total of 16 conditions, *P*_1_ = 0.5 (left) to 1.0 (right) with *P*_*DEG*_ = 0.45 (top) to 0.75 (bottom). MBCdeg1 and MBCdeg2 were used for analysis with *K* = 3. The performance of MBCdeg2 partly depended on the of DEGES normalization (see Tables 2 and 3)

The results described above were obtained with *P*_*DEG*_ ≤ 0.25 and *K* = 3, and it may be reasonable to expect the largest cluster to consist of many non-DEGs for *P*_*DEG*_ < 0.5. However, there may be instances where *P*_*DEG*_ > 0.5 [37, 42], and we investigated the effect of *P*_1_ = 0.5, 0.7, 0.9, and 1.0 on larger *P*_*DEG*_ values (= 0.45–0.75) (Fig. 3). Our results showed that MBCdeg gave high AUC values in most trials with *P*_*DEG*_ ≤ 0.55 and *P*_1_ ≤ 0.9. However, its performance was decreased drastically in other conditions (e.g., *P*_1_ ≥ 0.9 and *P*_*DEG*_ ≥ 0.65).

Overall, the performance of MBCdeg was similar to TCC, and there were few differences between the performance of MBCdeg1 and MBCdeg2. However, MBCdeg1 is superior to both TCC and MBCdeg2 for *P*_*DEG*_ = 0.75 and *P*_1_ = 0.7. This may be explained by the failure of DEGES normalization that was used in TCC and MBCdeg2. The DEGES normalization works well in the conditions where *P*_*DEG*_ ≤ 0.45 and *P*_1_ ≤ 1.0, *P*_*DEG*_ ≤ 0.55 and *P*_1_ ≤ 0.9, *P*_*DEG*_ ≤ 0.65 and *P*_1_ ≤ 0.7, and *P*_*DEG*_ ≤ 0.75 and *P*_1_ ≤ 0.5. However, the performance of the normalization decreases in other conditions, and these results are similar to those reported by Evans et al. [35].

Table 2 shows a representative result for MBCdeg for *P*_*DEG*_ = 0.75, and *P*_1_ = 0.7. In this condition, 5250 DEG1, 2250 DEG2, and 2500 non-DEG patterns were observed. MBCdeg1 gave three clusters, each of which consisted of many genes that resembled one of the three patterns, with a particular pattern predominating. In the trial shown in Table 2a, MBCdeg1 successfully identified the third cluster displaying the minimum norm value (= 0.3145) as the non-DEG cluster and produced an accurately ranked gene list with a high AUC value (= 0.9329). However, in the same trial shown in Table 2b, MBCdeg2 incorrectly determined the third cluster displaying the minimum norm value (= 0.0762) as the non-DEG cluster and produced an inaccurately ranked gene list with a low AUC value (= 0.2421). As the third cluster mainly consists of genes with the DEG1 pattern (4942/5637 = 87.7%), genes that belong to the first and second clusters will be located at the top of the ranked gene list. The AUC value (= 0.2421) may result due to genes in the first cluster showing predominantly DEG2 patterns (1979/2436 = 81.2%) that are correctly ranked at the top.

**Table 2:** Representative results for MBCdeg with *P*_*DEG*_ = 0.75 and *P*_1_ = 0.7

	DEG1	DEG2	non-DEG	Total	*L*^*2*^ Norm
(a) MBCdeg1 (AUC = 0.9329)					
First cluster	113	1971	345	2429	1.3039
Second cluster	4941	140	557	5638	0.6636
Third cluster	196	139	1598	1933	0.3145
(b) MBCdeg2 (AUC = 0.2421)					
First cluster	113	1979	344	2436	1.8909
Second cluster	195	132	1600	1927	0.9017
Third cluster	4942	139	556	5637	0.0762
Truth	5250	2250	2500	10,000	

The results for (a) MBCdeg1 and (b) MBCdeg2 in the same trial as described in Fig. 3 are shown. The results indicate that MBCdeg1 outperforms MBCdeg2

In the scenario where MBCdeg1 is inferior to MBCdeg2, we observed 22% of trials (= 11/50) with extremely low AUC values (< 0.2) for MBCdeg1 with *P*_*DEG*_ = 0.45 and *P*_*1*_ = 1.0. As this condition has 4500 DEG1 and 5500 non-DEG patterns, the accurate number of clusters is two (i.e., *K*_*truth*_ = 2). Table 3 shows the representative case of an MBCdeg trial with *K* = 3. We observed that MBCdeg1 incorrectly determined the third cluster that displayed the minimum norm value (0.4100) as non-DEG one and produced an inaccurately ranked gene list with an extremely low AUC value (= 0.1195). As this condition does not contain any DEG2 pattern, the failure to identify non-DEG cluster results in a non-ideal and incorrectly ranked gene list. This produces several extremely inferior results (11/50 = 22% of trials) when using MBCdeg1. A greater number of successful trials using MBCdeg1 (39/50 = 78% of trials) with *P*_*DEG*_ = 0.45 and *P*_*1*_ = 1.0 may result due to the number of non-DEG patterns being greater than DEG1 patterns (i.e., 55:45). Similar explanations for the fewer successful cases using MBCdeg with *P*_*DEG*_ = 0.55 and *P*_1_ = 1.0 may be valid as the number of non-DEG patterns is fewer than the number of DEG1 patterns (i.e., 45:55). MBCdeg2 shows stable and relatively accurate performance for *P*_*DEG*_ ≤ 0.45, regardless of the value of *P*_1_ and it may be useful in these conditions. This trend was also observed when the number of replicates per group was increased to *n*_1_ = *n*_2_ = 6, 9, and 12 (Additional file 5).

**Table 3:** Results for MBCdeg with *P*_*DEG*_ = 0.45 and *P*_1_ = 1.0

	DEG1	DEG2	non-DEG	Total	*L*^2^ Norm
(a) MBCdeg1 (AUC = 0.1195)					
First cluster	3269	0	391	3660	0.5717
Second cluster	788	0	5081	5869	0.4541
Third cluster	443	0	28	471	0.4100
(b) MBCdeg2 (AUC = 0.9547)					
First cluster	3894	0	589	4483	0.8698
Second cluster	22	0	3	25	3.0380
Third cluster	584	0	4908	5492	0.1118
Truth	4500	0	5500	10,000	

The results for (a) MBCdeg1 and (b) MBCdeg2 in the same trial as described in Fig. 3 are shown. The results indicate that MBCdeg2 outperforms MBCdeg1

### Three-group simulated data

Next, we investigated the performance of MBCdeg in a multi-group comparison. Tang et al. [43] evaluated the performance of 12 DE pipelines available across nine R packages (TCC [14], edgeR [11], DESeq [44], DESeq2 [15], limma [45], samr [46], PoissonSeq [47], baySeq [12], and EBSeq [13]) and recommended the use of TCC when performing three-group count data. Therefore, we followed the conditions where TCC performed the best in the previous simulation analysis and investigated whether MBCdeg could exceed TCC as well as the other two methods (i.e., edgeR and DESeq2).

As in the evaluation study described [43], we focused on a three-group data with equal numbers of replicates (i.e., three replicates per group; *n*_1_ = *n*_2_ = *n*_3_ = 3). The other simulation conditions were *G* = 10,000, *P*_*DEG*_ = 0.25, and *FC* = 4. For the proportion of upregulated DEGs in individual groups (*P*_1_, *P*_2_, *P*_3_), we prepared a total of four conditions: (1/3, 1/3, 1/3), (0.6, 0.2, 0.2), (0.5, 0.5, 0.0), and (1.0, 0.0, 0.0). The numbers of true clusters *K*_*truth*_ for these conditions were 4, 4, 3, and 2, respectively. The first two conditions are the same as those used in the previous study, and the other two conditions were used to examine the effect on different *K*_*truth*_ values (i.e., 3 and 2). We compared a total of four *K* values (*K* = 2, 3, 4, and 5) when performing an analysis using MBCdeg. For the three packages (edgeR, DESeq2, and TCC), gene ranking was performed based on an ANOVA-like *p*-value, where a low *p*-value for a gene indicates a high degree of DE in at least one of the groups compared.

**Fig. 4:**
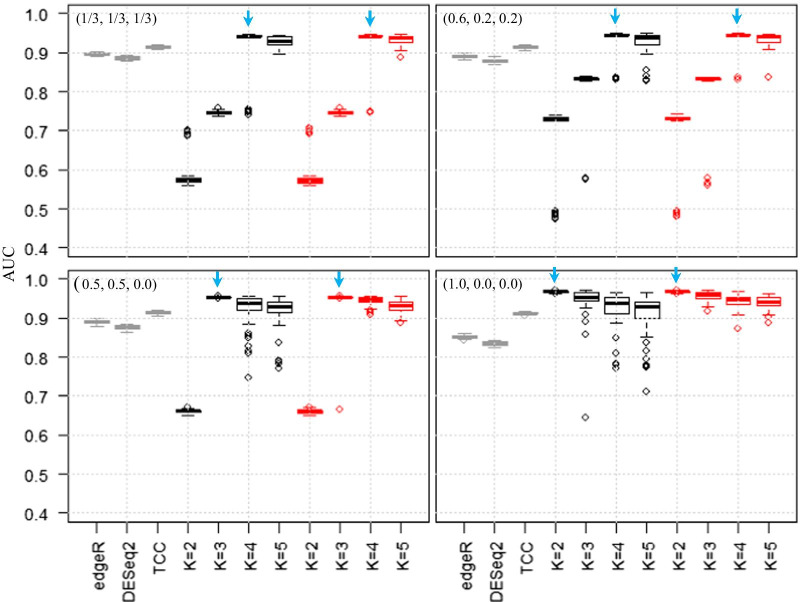
Results for three-group simulated data. Boxplots of AUC values (50 trials) for eleven methods (DESeq2, edgeR, TCC, and MBCdeg with *K* = 2–5) are shown. The simulation conditions used were *P*_*DEG*_ = 0.25, *FC* = 4, and *n*_1_ = *n*_2_ = *n*_3_ = 3

Figure 4 shows the boxplots of AUC values using these methods in the four conditions described. We observed that the AUC values were the highest when *K* = *K*_*truth*_ and that the performances with *K* > *K*_*truth*_ were higher than those with *K* < *K*_*truth*_. Further, this trend was observed when the number of replicates per group was increased to *n*_1_ = *n*_2_ = *n*_3_ = 6, 9, and 12 (Additional file 6). However, for simulated data with variable *FC* values, AUC values for MBCdeg with *K* ≥ *K*_*truth*_ tended to be higher than those with *K* = *K*_*truth*_ (Additional file 7). This is consistent with the results of the two-group data (Additional file 4). Together, these results indicate that MBCdeg yields better performance with larger *K* values (e.g., *K* > *I* + 1) when using real data with a group number *I*.

### Two-group real data

We examined the performance of MBCdeg for a real count dataset available from the recount2 database [48]. As with previous analyses, we compared a total of five methods: edgeR, DESeq2, TCC, MBCdeg1, and MBCdeg2. For the first three packages, genes with a false discovery rate (FDR) of 0.1 or higher were defined as non-DEGs. The other genes were identified as having DEG1 or DEG2 patterns in the direction of DE. Some may argue that this cut-off (10% FDR) is somewhat looser than the commonly used ones (e.g., 1% or 5%). However, we empirically know that even with such a loose threshold of 10% FDR, the number of genes satisfying the threshold will be lower than the true number of DEGs [20]. Hence, we decided that it is more reasonable to use the default TCC threshold of 10% FDR for the sake of comparison in the context of the MBCdeg results. For MBCdeg, we investigated the effect on different *K* values (= 3, 4, and 5). As previously described, genes belonging to the cluster with the smallest *L*^2^ Norm were considered as non-DEGs. The other genes were identified as having DEG1 or DEG2 patterns in the direction of DE, regardless of the cluster to which the genes belonged. As the true DE results are not known, we focus our attention on the similarities between the methods.

The real dataset (called “Pickrell”) consisting of 51,910 × 69 samples was designed to compare the expression levels of lymphoblastoid cell lines between 40 females and 29 males [49]. This dataset has been widely used for validation purposes [50]. Table 4 shows the results of the gene classification into one of the three patterns. Overall, MBCdeg1 with *K* = 5 showed (i) the maximum *P*_*DEG*_ value of (1291 + 3433)/51,910 = 0.0910, and (ii) the maximum degree of asymmetry, defined as max(*P*_1_, *P*_2_), of 3433/(1291 + 3433) = 0.7267. These values are within the range used in the simulation analysis (see Additional files 2 and 4). The values in parentheses indicate the average log_2_(*FC*) values for the genes. Our results showed that the values for DEG1 and DEG2 are approximately 1.8 (i.e., the average *FC* is about 2^1.8 = 3.5), which is within the values of *FC* = 2.5 and 4 introduced in the current simulation analysis.

**Table 4:** DE results using the Pickrell data

Method	DEG1	DEG2	Non-DEG
edgeR	1675 (− 1.677)	2872 (1.911)	47,363 (0.074)
DESeq2	66 (− 1.520)	52 (4.083)	51,792 (0.117)
TCC	65 (− 1.935)	123 (3.695)	51,722 (0.113)
MBCdeg1 (*K* = 3)	1291 (− 1.742)	3298 (1.800)	47,321 (0.053)
MBCdeg1 (*K* = 4)	1291 (− 1.742)	3393 (1.894)	47,226 (0.043)
MBCdeg1 (*K* = 5)	1291 (− 1.742)	3433 (1.889)	47,186 (0.041)
MBCdeg2 (*K* = 3)	1291 (− 1.742)	3209 (1.802)	47,410 (0.056)
MBCdeg2 (*K* = 4)	1351 (− 1.694)	3219 (1.802)	47,340 (0.057)
MBCdeg2 (*K* = 5)	1329 (− 1.715)	3302 (1.907)	47,279 (0.046)

The number of genes and average log_2_(*FC*) values in each pattern are shown. The *FC* is defined as the mean normalized count of samples in group 2 (male group) divided by the mean normalized count of samples in group 1 (female group). To evaluate the differences in the genes assigned to each pattern between methods, the log_2_(*FC*) values for individual genes obtained by edgeR were used for the other methods

DESeq2 and TCC showed similar results (99.8% concordance), and edgeR and MBCdeg also showed similar results (> 97% concordance). Even the lowest concordance rate was found between DESeq2 and MBCdeg1 with *K* = 5 at 90.86% (see “Sheet1” in Additional file 8). This high concordance rate (≥ 90.86%) among the methods is reasonable as the maximum *P*_*DEG*_ value is 9.10% (i.e., mostly determined as non-DEGs). We observed a high similarity between the results from six MBCdeg methods, where the lowest concordance rate was found between MBCdeg1 with *K* = 5 and MBCdeg2 with *K* = 4 at 97.26%. This suggests that a cluster of DEG1 (or DEG2) patterns obtained by *K* = 3 is divided into sub-clusters when *K* = 4 or 5. Indeed, MBCdeg1 with *K* = 4 produced two clusters (containing 1939 genes and 1454 genes, respectively) assigned to the DEG2 pattern (see “Sheet2” in Additional file 8). Of a total of 3393 genes, 2713 genes were included in the 3298 DEG2 genes obtained by MBCdeg1 with *K* = 3 (Table 4).

Similar to the analysis using MBCdeg1 with *K* = 4, MBCdeg2 with *K* = 4 produced two clusters (containing 60 genes and 1291 genes, respectively) assigned to the DEG1 pattern (“Sheet2” in Additional file 8). Interestingly, the 1291 genes in the second cluster were identical to the 1291 DEG1 genes obtained using MBCdeg2 with *K* = 3, and 1290 of the 1291 genes were the same as those obtained using MBCdeg1 with *K* = 3–4. In the results obtained using MBCdeg2 with *K* = 4, we found that the representative expression patterns for the remaining 60 genes assigned to the DEG1 pattern (the cluster centers *μ* = (0.184, − 0.184)) were very similar to those assigned to the non-DEG pattern (*μ* = (0.014, − 0.014)) than the other 1291 genes to the DEG1 pattern (*μ* = (3.117, − 3.117)), given the degrees of DE. These 60 genes were assigned to non-DEG pattern at *K* = 3. Although we defined only one cluster with the smallest *L*^2^ Norm as of non-DEG, it may be better to define the clusters with low *L*^2^ Norm as of non-DEGs and to consider their assignments when using other *K* values.

In the results with *K* = 5, we observed that MBCdeg2 gave two DEG1 clusters containing 38 and 1291 genes, and two DEG2 clusters containing 1938 and 1364 genes. Additionally, MBCdeg1 gave one DEG1 cluster containing 1291 genes and three DEG2 clusters containing 1923, 39, and 1471 genes, respectively. As described earlier, the 1291 DEG1 genes obtained by MBCdeg2 with *K* = 5 were identical to those obtained by MBCdeg2 with *K* = 3–4 and 1290 out of the 1291 DEG1 genes were the same as those obtained by MBCdeg1 with *K* = 3–5. The degrees of DE in the three DEG2 clusters obtained when using MBCdeg1 were sorted in descending order of the clusters consisting of 1923, 39, and 1471 genes (“Sheet2” in Additional file 8). The 1962 genes from the first two DEG2 clusters were included in the DEG2 cluster containing 3298 genes, that was obtained by using MBCdeg1 with *K* = 3. However, 751 (or 720) of the remaining 1471 genes with the lowest degree of DE from the last DEG2 cluster, were included alone in the DEG2 (or non-DEG) cluster obtained by using MBCdeg1 with *K* = 3. These results suggest that the use of MBCdeg for DE analysis should take into account the fluctuation of expression patterns in clusters obtained at various *K* values, as well as the PPs assigned to each gene.

## Discussion

Gene clustering has been used to classify DEGs with similar expression patterns. The MBCluster.Seq package was originally developed to be used as a post-DE analysis tool. In this study, we propose the use of this model-based gene clustering algorithm for DE analysis itself (i.e., the identification of DEGs). Although the creators of MBCluster.Seq have not envisioned this possibility, the DE framework based on the package (called MBCdeg) outperformed three other packages (edgeR, DESeq2, and TCC) dedicated to DEG identifications in many DE scenarios with *P*_*DEG*_ < 50%.

Several bioinformatics studies have made comparisons with typical conventional analysis methods and claimed about superior results by using the methods of interest [51]. However, a typical package may not be the best suited for the analysis. As demonstrated in this study, the less well-known TCC package gave better results in our analysis than the typical packages (edgeR and DESeq2). The main contribution of this study is the demonstration that clustering-based DE framework (MBCdeg) performed better than TCC under the scenarios for which TCC performed well.

MBCdeg2 (MBCdeg with robust DEGES normalization) was slightly more stable and accurate than MBCdeg1 (MBCdeg with the default normalization) when using the simulation analysis employed in our study. A common disadvantage of the MBCdeg is that in datasets with a very large proportion of DEGs (*P*_*DEG*_ ≥ 50%), the identification of the non-DEG cluster, which is the key to the framework proposed here, fails frequently and results in incorrect rankings. Therefore, conventional methods must be used in conjunction with MBCdeg to ensure that the overall similarities in the rankings are maintained. Additionally, it is necessary to verify various *K* values to investigate the cluster-wise fluctuations for DEGs and the number of clusters identified as optimal by MBCdeg. While the current simulation analysis was done with TCC, there are several other simulation frameworks available (e.g., polyester [52] and countsimQC [53]). The proposed method requires (i) evaluation based on those simulation frameworks, (ii) real data with various experimental settings and organisms, and (iii) additional fine-tuning described above.

## Conclusions

The model-based gene clustering allowed by the MBCluster.Seq package is a promising tool for both the identification and classification of DEGs in one step. The proposed procedure, MBCdeg, can be used in the context of RNA-seq count data in which the percentage of DEGs is less than half (*P*_*DEG*_ < 50%).

## Methods

All analyses were performed using CRAN/R (ver. 3.6.3) [54] and Bioconductor [55]. The versions of the major R packages used were MBCluster.Seq ver. 1.0 [28], TCC ver. 1.26.0 [14], edgeR ver. 3.28.1 [11], DESeq2 ver. 1.26.0 [15], ROC ver. 1.6.3, and recount ver. 1.12.1. The R-codes leading to the results described in this paper are available on GitHub (https://github.com/takosa/MBCdeg-paper). A two-group sample data and R-codes (MBCdeg1 and MBCdeg2) for data execution are also provided.

### Simulated data

The simulated data for two- and three-group comparisons were generated using the *simulateReadCounts* function in TCC [14]. The number of genes (*G* = 10,000) was given in the *Ngene* option. The number of replicates for individual groups (i.e., *n*_1_, *n*_2_, …, *n*_*I*_ for *I*-group comparison) were specified in the *replicates* option. The proportion of DEGs (*P*_*DEG*_) was given in the *PDEG* option. The assignment of DEGs upregulated in individual groups (i.e., *P*_1_, *P*_2_, …, *P*_*I*_, and *P*_1_ + *P*_2_ + … + *P*_*I*_ = 1) was specified in the *DEG.assign* option. The fixed degrees of DE (*FC* = 4) were specified in the *DEG.foldchange* option. To generating different degrees of DE, the *makeFCMatrix* function was used, and the output object was used as the input for the *fc.matrix* option in the *simulateReadCounts* function. The output of the *simulateReadCounts* function was stored in the TCC class object with information about the simulation conditions and is therefore ready-to-analyze in the DE analysis.

### Real data

Pickrell’s real data was obtained by searching the recount2 database with the accession number “SRP001540”. The original count matrix (58,037 genes × 160 samples) consists of human data obtained from two different sequencing centers; one from Yale and the other from Argonne [48, 49]. Since both datasets have been found to show similar results [50], we analyzed the Yale count data of 79 samples alone. The matrix was collapsed by summing the data for technical replicates, resulting in a reduced number of columns/samples (79 → 69). Genes with a count of zero in all samples were excluded (58,037 → 51,910). DE analysis was performed using 40 female samples vs. 29 male samples.

### DE analysis

DE analysis using edgeR was performed by applying the following functions with the default options in the sequence indicated: *DGEList*, *calcNormFactors*, *estimateDisp*, *glmQLFit*, *glmQLFTest*, and *topTags*. The *p*-values for individual genes were obtained by the output of the *topTags* function. The genes were ranked in ascending order based on the *p*-values and the ranks were used to calculate the AUC values. The AUC values were calculated using the *AUC* function in the ROC package. The *p*-values were adjusted for multiple testing by using the Benjamini–Hochberg procedure [56]. The adjusted *p*-values (*q*-values) were obtained using the *p.adjust* function with the *method* = *”BH”* option. Genes that had a *q*-value greater than 0.1 were defined as having a non-DEG pattern. The log_2_(FC) values for individual genes were obtained from the output of the *topTags* function (the *logFC* column in the *table* slot). Genes that showed a *q*-value less than 0.1, and genes with log_2_(FC) values less (or greater) than 0 were defined as having a DEG1 (or DEG2) pattern.

DE analysis using DESeq2 [15] was performed by applying the following functions with default options in the sequence indicated: *DESeqDataSetFromMatrix*, *DESeq*, and *results*. The *p*-values for individual genes were obtained by using the output of the *results* function (the *pvalue* column). The adjusted *p*-values (*q*-values) were obtained from the output of the *results* function (the *padj* column). The other procedures followed were the same as those described in edgeR.

DE analysis using TCC was performed by using the following functions with default options in the sequence indicated: *new*, *calcNormFactors*, *estimateDE*, and *getResult*. The gene ranking information and *q*-values were obtained directly from the output of the *getResult* function. The other procedures were the same as those described in edgeR. The normalization factors assigned for individual samples were obtained from the output of the *calcNormFactors* function in TCC. The normalization factors were converted to size factors, and the log-transformed values were used in the *Normalizer* option in the *RNASeq.Data* function when using MBCdeg2.

The DE analysis based on the MBCluster.Seq method [28] (i.e., MBCdeg) was performed by applying the following functions in sequence: *RNASeq.Data*, *KmeansPlus.RNASeq*, and *Cluster.RNASeq*. The *Normalizer* = *NULL* option in the *RNASeq.Data* function was used when performing an analysis using MBCdeg1 as it corresponds to the default normalization algorithm [33] employed in MBCluster.Seq. The preselected number of clusters *K* was introduced in the *nK* option in the *KmeansPlus.RNASeq* function. The negative binomial model (“nbinom”) was used in the *model* option in both *KmeansPlus.RNASeq* and *Cluster.RNASeq* functions. The output of the *KmeansPlus.RNASeq* function was used as the initial cluster center when executing the *Cluster.RNASeq* function. The expectation–maximization (EM) algorithm was used to iteratively update the estimates of clusters and their centers. This corresponds to the *method* = *“EM”* option in the *Cluster.RNASeq* function. The centers for *K* clusters (*k* = 1, …, *K*) across *I* groups (*i* = 1, …, *I*), *μ*_*ki*_, were used to calculate the *L*^2^ Norm values ||*μ*_*k*_||_2_ for individual clusters. The information for cluster centers and the PPs for individual genes (*g* = 1, …, *G*) across clusters were obtained as the outputs of the *Cluster.RNASeq* function. Genes in a cluster that had the smallest *L*^2^ Norm value were regarded as non-DEGs. The other genes were determined to have DEG1 or DEG2 patterns in the direction of DE when performing a two-group comparison (*I* = 2). The overall gene ranking was performed based on the PPs assigned to the non-DEG cluster.

## Supplementary Information

**Additional file 1.** Results corresponding to Fig. 1 with a larger number of replicates. Boxplots of AUC values (100 trials) for individual methods with n1 = n2 = (a) 6, (b) 9, and (c) 12 are shown. In the simulation, the degree of DE was fixed at 4-fold (i.e., FC = 4).

**Additional file 2.** Results corresponding to Fig. 2 with a larger number of replicates. Boxplots of AUC values (100 trials) for MBCdeg (K = 2–4) with n1 = n2 = (a) 6, (b) 9, and (c) 12 are shown.

**Additional file 3.** Effect on different degrees of DE for the five methods. Boxplots of AUC values (100 trials) for individual methods with n1 = n2 = (a) 3, (b) 6, (c) 9, and (d) 12 are shown. In contrast to Fig. 1 and Additional file 1, simulations were performed using different degrees of DE.

**Additional file 4.** Effect on different degrees of DE for MBCdeg with K = 2–4. Boxplots of AUC values (100 trials) for MBCdeg (K = 2–4) with n1 = n2 = (a) 3, (b) 6, (c) 9, and (d) 12 are shown. In contrast to Fig. 2 and Additional file 2, simulations were performed using different degrees of DE. The AUC values for MBCdeg with K = 3 were almost the same as those in Additional file 3 (different trials were used).

**Additional file 5.** Results corresponding to Fig. 3 with a larger number of replicates. Boxplots of AUC values (50 trials) for individual methods with n1 = n2 = (a) 6, (b) 9, and (c) 12 are shown.

**Additional file 6.** Results corresponding to Fig. 4 with a larger number of replicates. Boxplots of AUC values (50 trials) for individual methods with n1 = n2 = n3 = (a) 6, (b) 9, and (c) 12 are shown.

**Additional file 7.** Results corresponding to Additional file 4 on the three-group simulated data. Boxplots of AUC values (50 trials) for individual methods with n1 = n2 = n3 = (a) 3, (b) 6, (c) 9, and (d) 12 are shown.

**Additional file 8.** Concordance rate between methods for Pickrell data. This file provides additional information given in Table 4. The “Sheet1” shows the percentage of genes assigned to the same pattern that are common between the two methods. For example, DESeq2 and TCC have a total of 51,812 similar patterns, and the concordance rate is therefore calculated as 51,812/51,910 = 0.9981. The “Sheet2” shows the results for each cluster obtained using MBCdeg-based methods: (a–c) MBCdeg1 with K = 3–5 and (d–f) MBCdeg2 with K = 3–5. The number of genes (column B), centers m (columns C–E), L2 Norm (column F), and the patterns assigned (column G) for each cluster are shown.

## Data Availability

The dataset (SRP001540) analyzed during the current study is available in the recount2 website, [https://jhubiostatistics.shinyapps.io/recount/]. The R-codes leading to the results described in this paper are available on GitHub, [https://github.com/takosa/MBCdeg-paper]. A two-group sample data and R-codes (MBCdeg1 and MBCdeg2) for data execution are also provided.

## Abbreviations

ANOVA: Analysis of variance
AUC: The area under the receiver operating characteristic curve
*β*_*gi*_: Count of gene *g* in group *i* relative to the overall mean on log-scale
DE: Differential expression
DEG: Differentially expressed gene
DEG1: Expression pattern up-regulatd in group 1
DEG2: Expression pattern up-regulatd in group 2
EM: Expectation-maximization
FC: (Degree of) fold-change
G: Number of genes
GUI: Graphical user interface
*I*: Number of compared groups
*K*: Preselected number of clusters
*K*_*truth*_: True number of clusters
MBCdeg: The proposed DEG identification procedure based-on gene clustering with MBCluster.Seq
MBCdeg1: MBCdeg with default normalization algorithm
MBCdeg2: MBCdeg with a robust normalization algorithm called DEGES
*μ*_*k*_: Center for cluster *k*
*n*_*i*_: Number of replicates for group *i*
non-DEG: No differential expression pattern between groups
*p*_*gk*_: Posterior probability (PP) of gene *g* belonging to the *k*th cluster
*P*_*i*_: Proportion of up-regulated DEGs in group *i*
*P*_*DEG*_: Proportion of DEG
PP: Posterior probability
ROC: Receiver operating characteristic
