# Supplementary figures and images for: Differential expression analysis using a model-based gene clustering algorithm for RNA-seq data
Source: BMC Bioinformatics. 2021 Oct 20;22:511. doi: 10.1186/s12859-021-04438-4 (PMC8527798; doi:10.1186/s12859-021-04438-4)

## Slide 1
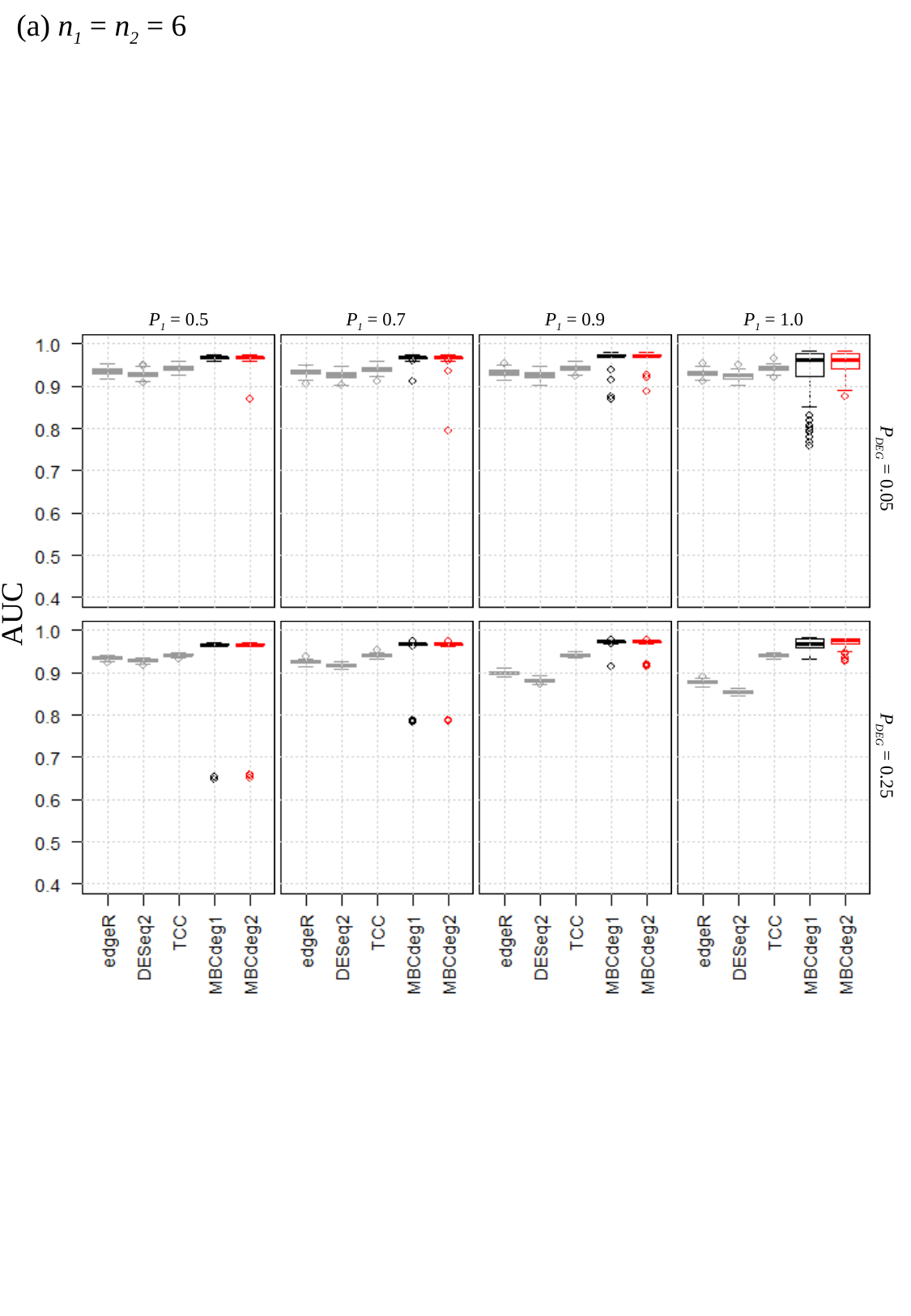

(a) n1 = n2 = 6
P1 = 0.5
P1 = 0.7
P1 = 0.9
P1 = 1.0
PDEG = 0.05
AUC
PDEG = 0.25

## Slide 2
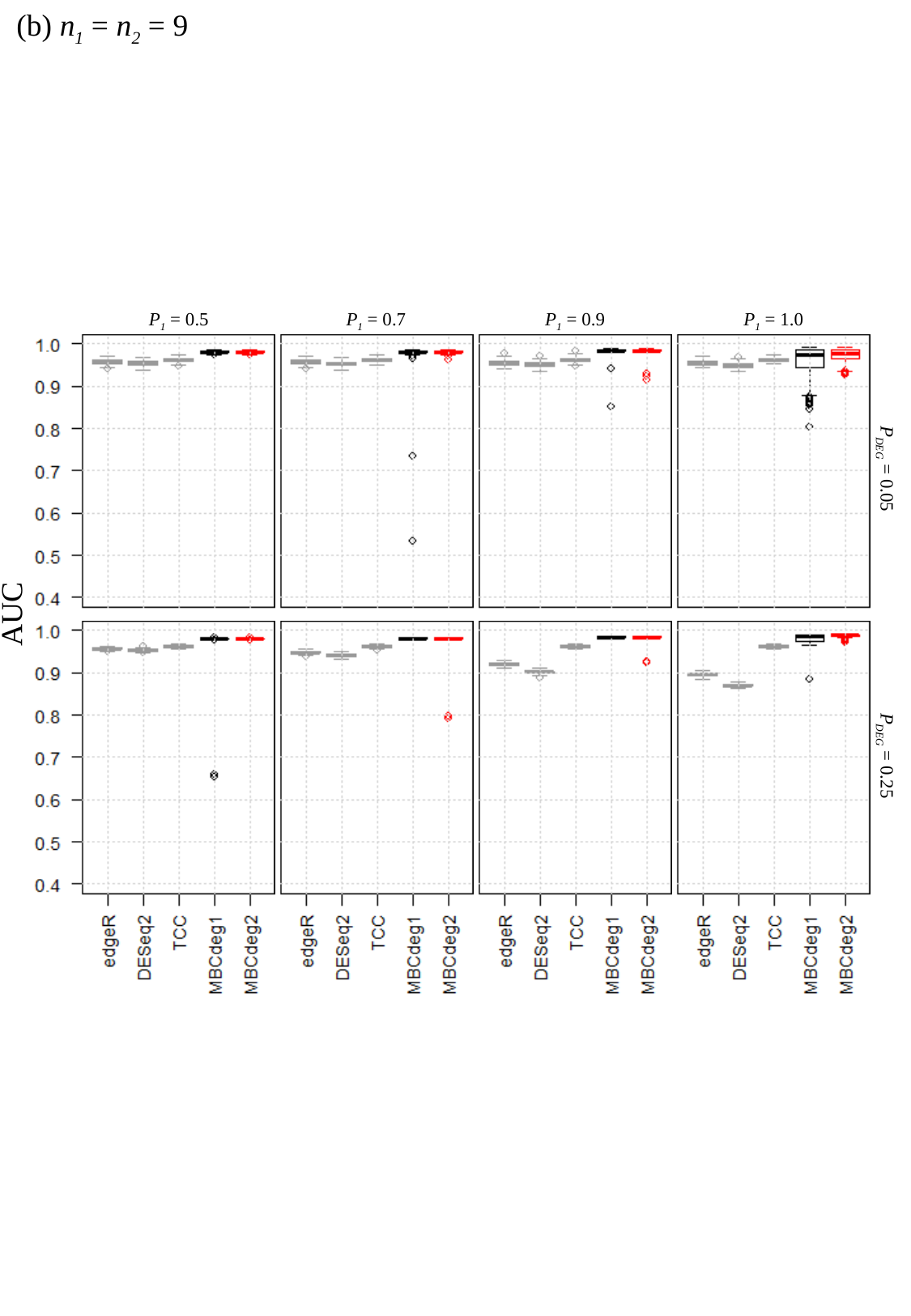

(b) n1 = n2 = 9
P1 = 0.5
P1 = 0.7
P1 = 0.9
P1 = 1.0
PDEG = 0.05
AUC
PDEG = 0.25

## Slide 3
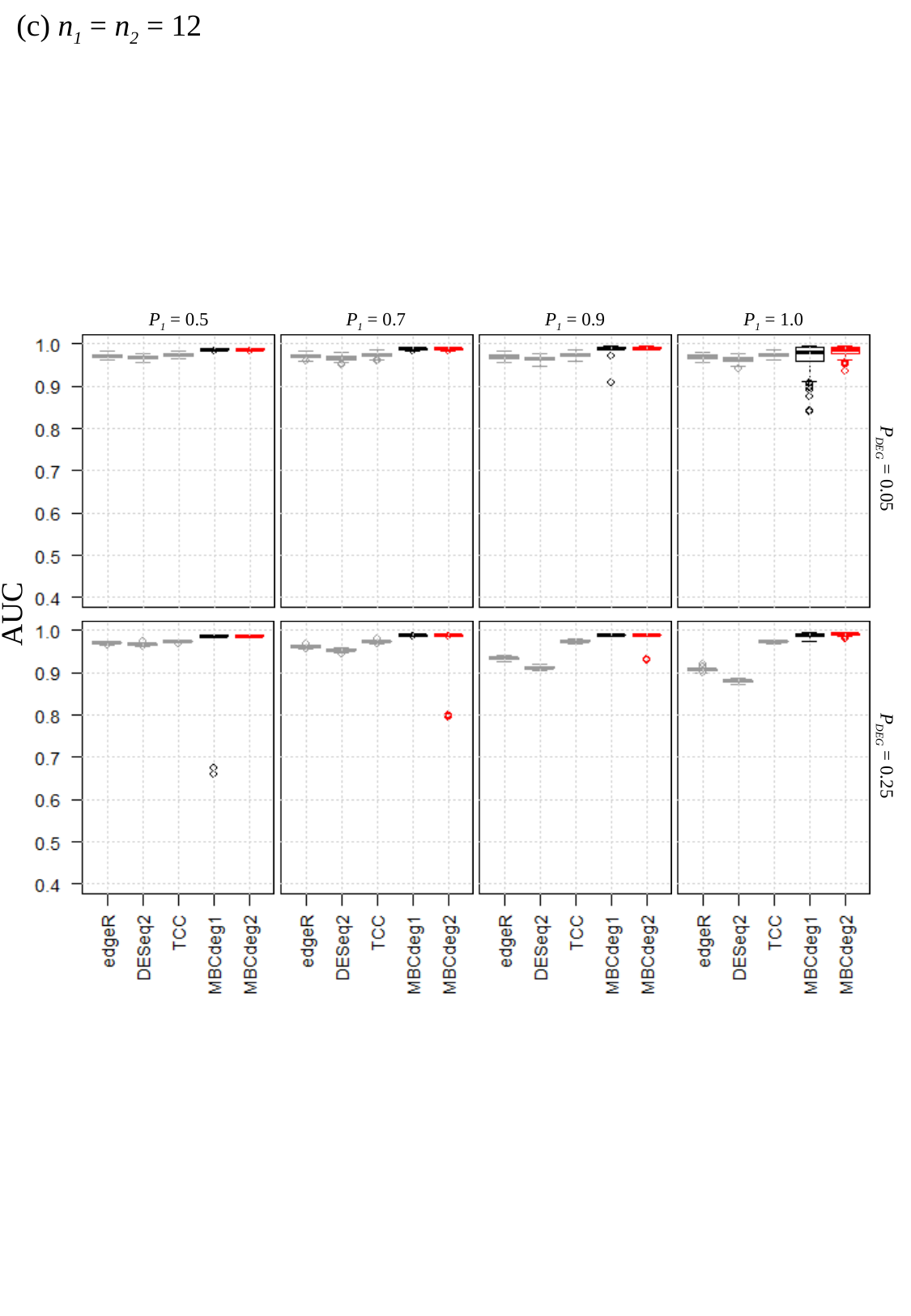

(c) n1 = n2 = 12
P1 = 0.5
P1 = 0.7
P1 = 0.9
P1 = 1.0
PDEG = 0.05
AUC
PDEG = 0.25

Supplement: Supplementary file 1 — Additional file 1. Results corresponding to Fig. 1 with a larger number of replicates. Boxplots of AUC values (100 trials) for individual methods with n1 = n2 = (a) 6, (b) 9, and (c) 12 are shown. In the simulation, the degree of DE was fixed at 4-fold (i.e., FC = 4). [file 12859_2021_4438_MOESM1_ESM.pptx]

## Slide 1
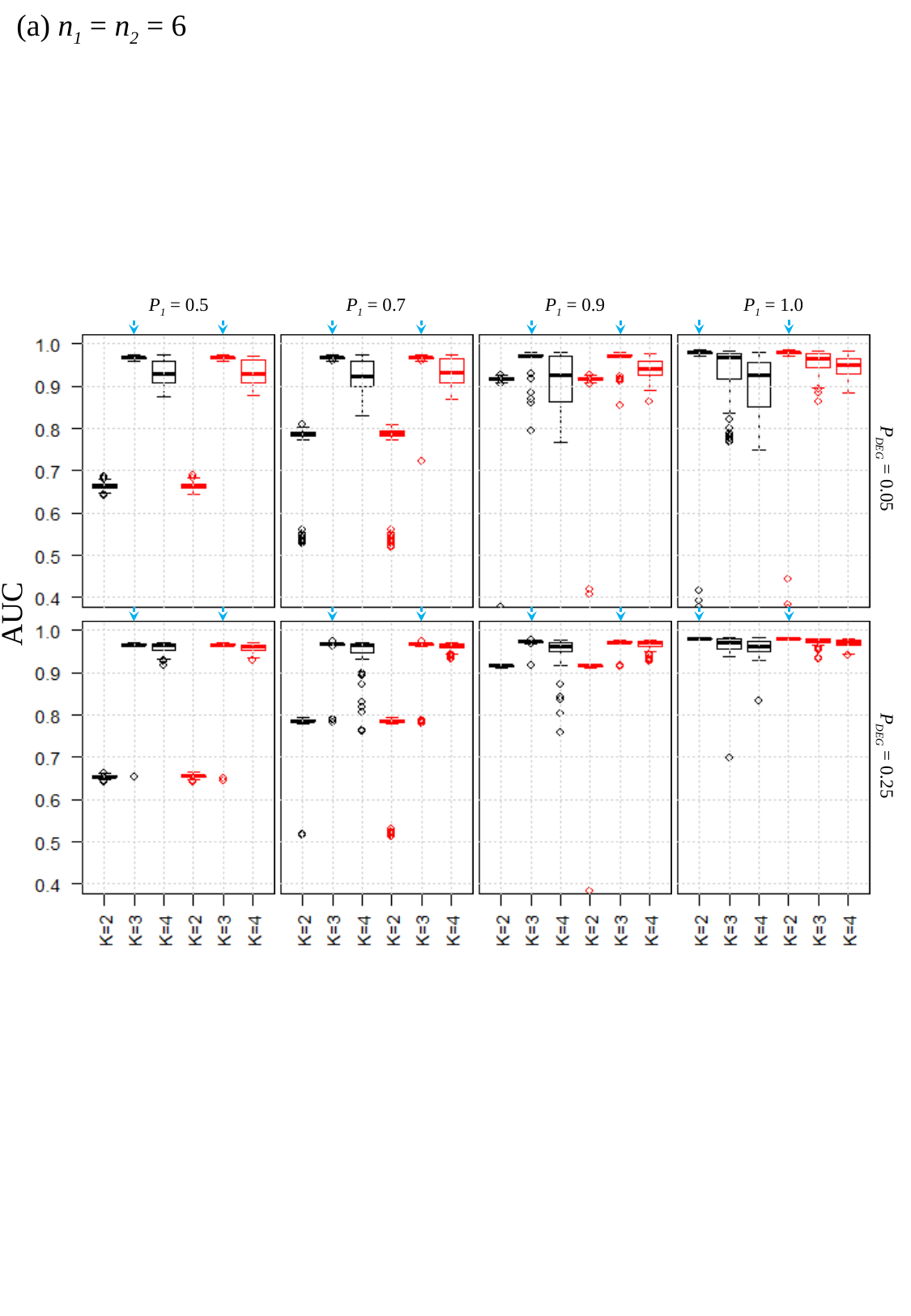

(a) n1 = n2 = 6
P1 = 0.5
P1 = 0.7
P1 = 0.9
P1 = 1.0
PDEG = 0.05
AUC
PDEG = 0.25

## Slide 2
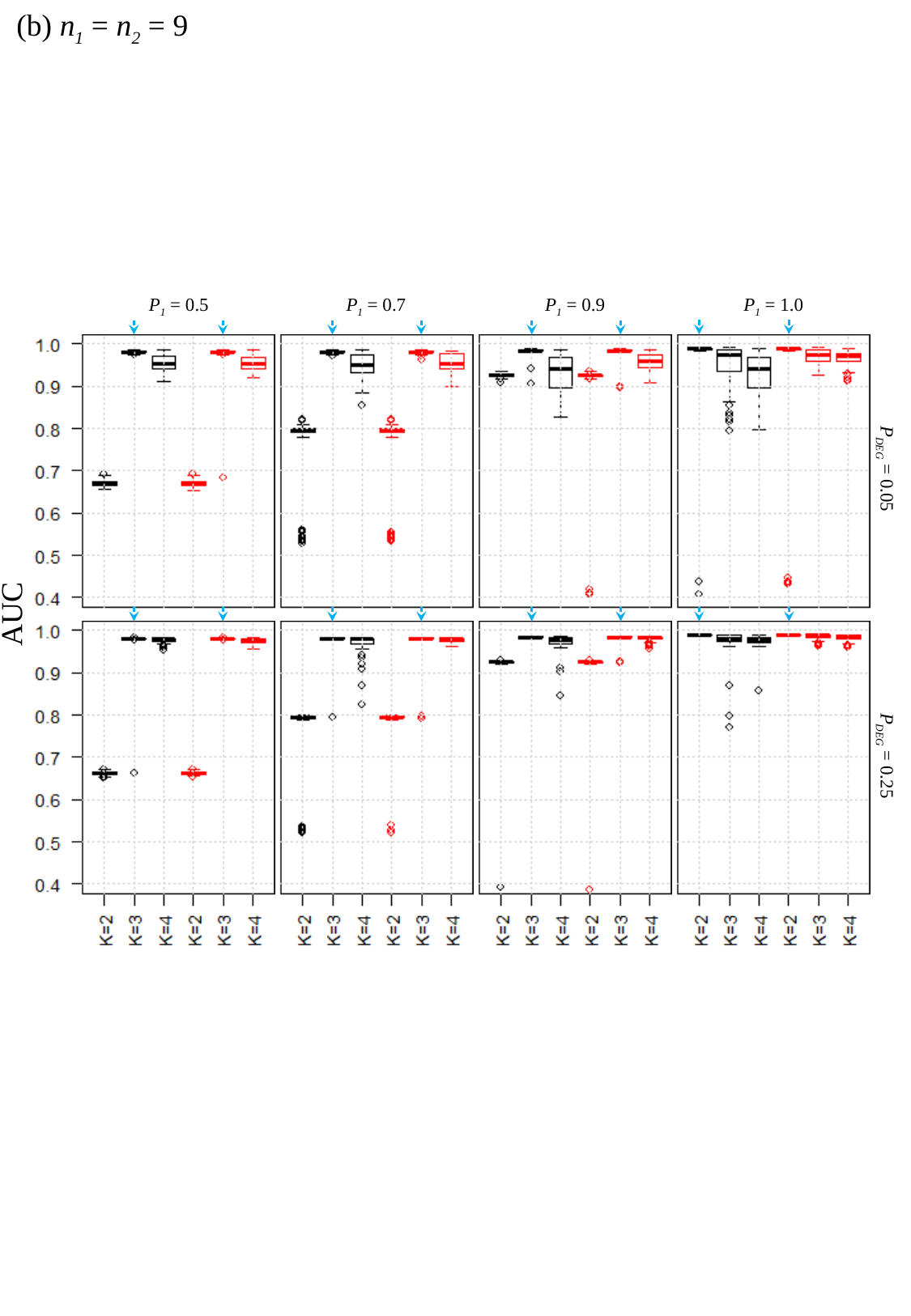

(b) n1 = n2 = 9
P1 = 0.5
P1 = 0.7
P1 = 0.9
P1 = 1.0
PDEG = 0.05
AUC
PDEG = 0.25

## Slide 3
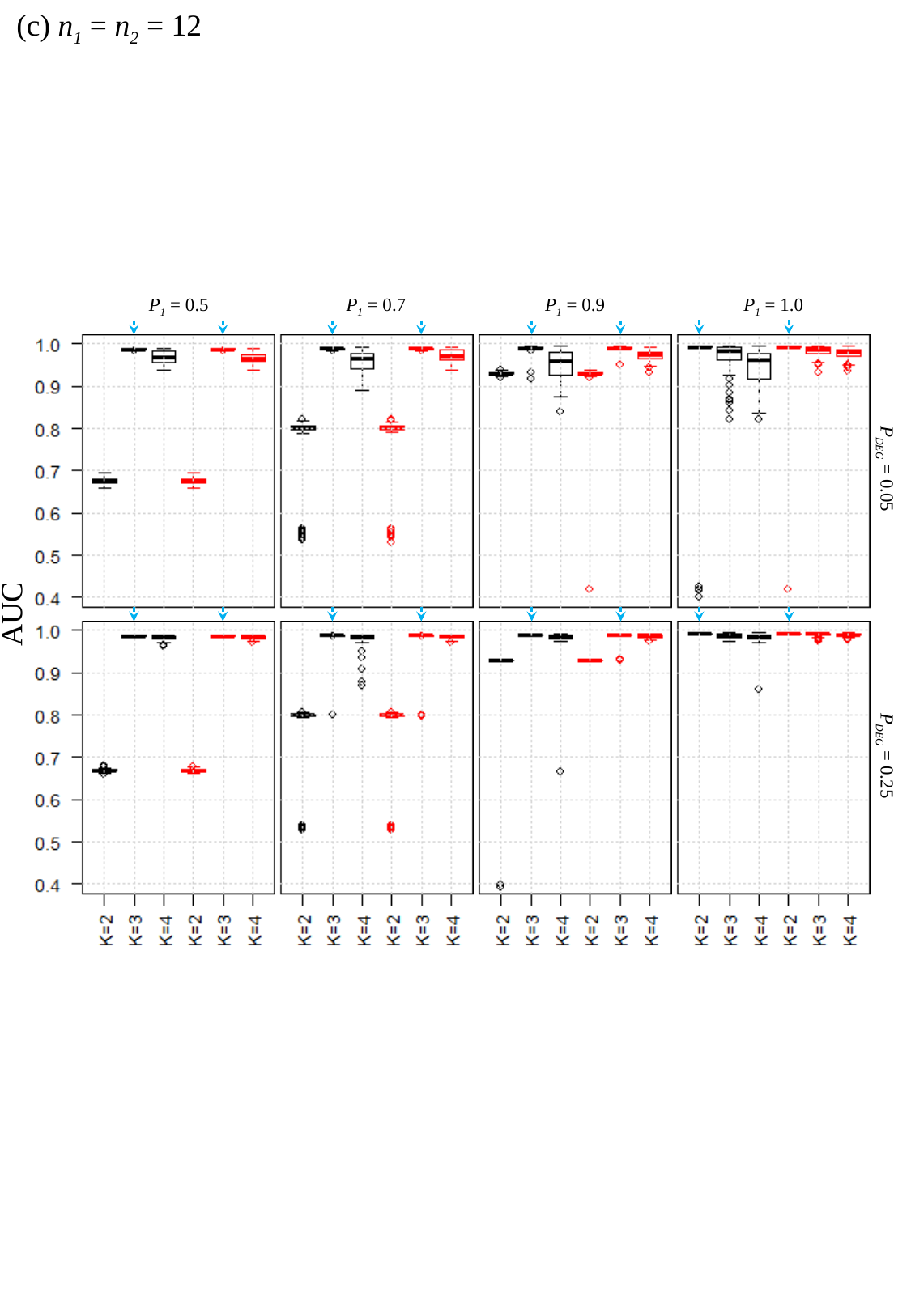

(c) n1 = n2 = 12
P1 = 0.5
P1 = 0.7
P1 = 0.9
P1 = 1.0
PDEG = 0.05
AUC
PDEG = 0.25

Supplement: Supplementary file 2 — Additional file 2. Results corresponding to Fig. 2 with a larger number of replicates. Boxplots of AUC values (100 trials) for MBCdeg (K = 2–4) with n1 = n2 = (a) 6, (b) 9, and (c) 12 are shown. [file 12859_2021_4438_MOESM2_ESM.pptx]

## Slide 1
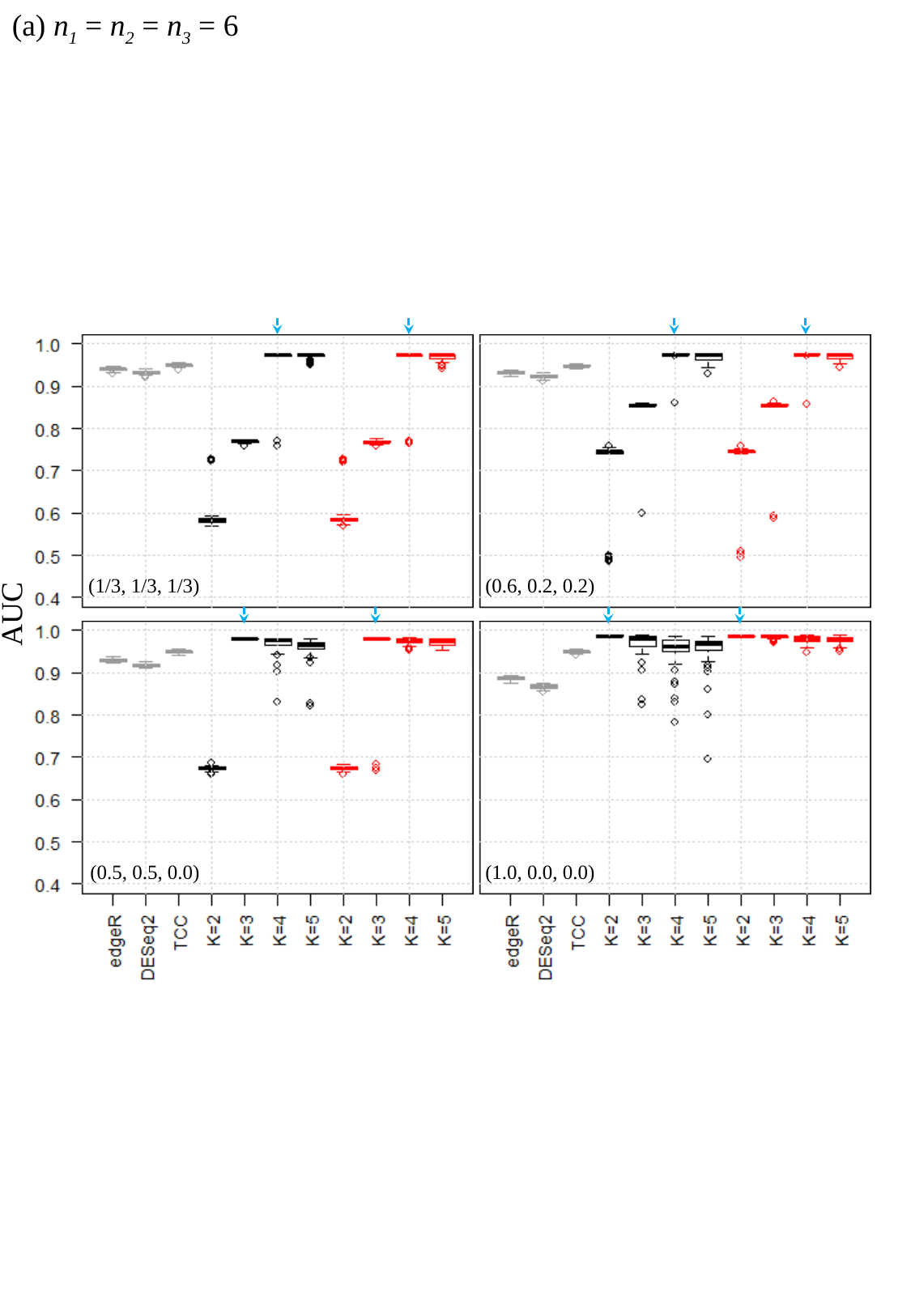

(a) n1 = n2 = n3 = 6
(1/3, 1/3, 1/3)
(0.6, 0.2, 0.2)
AUC
(0.5, 0.5, 0.0)
(1.0, 0.0, 0.0)

## Slide 2
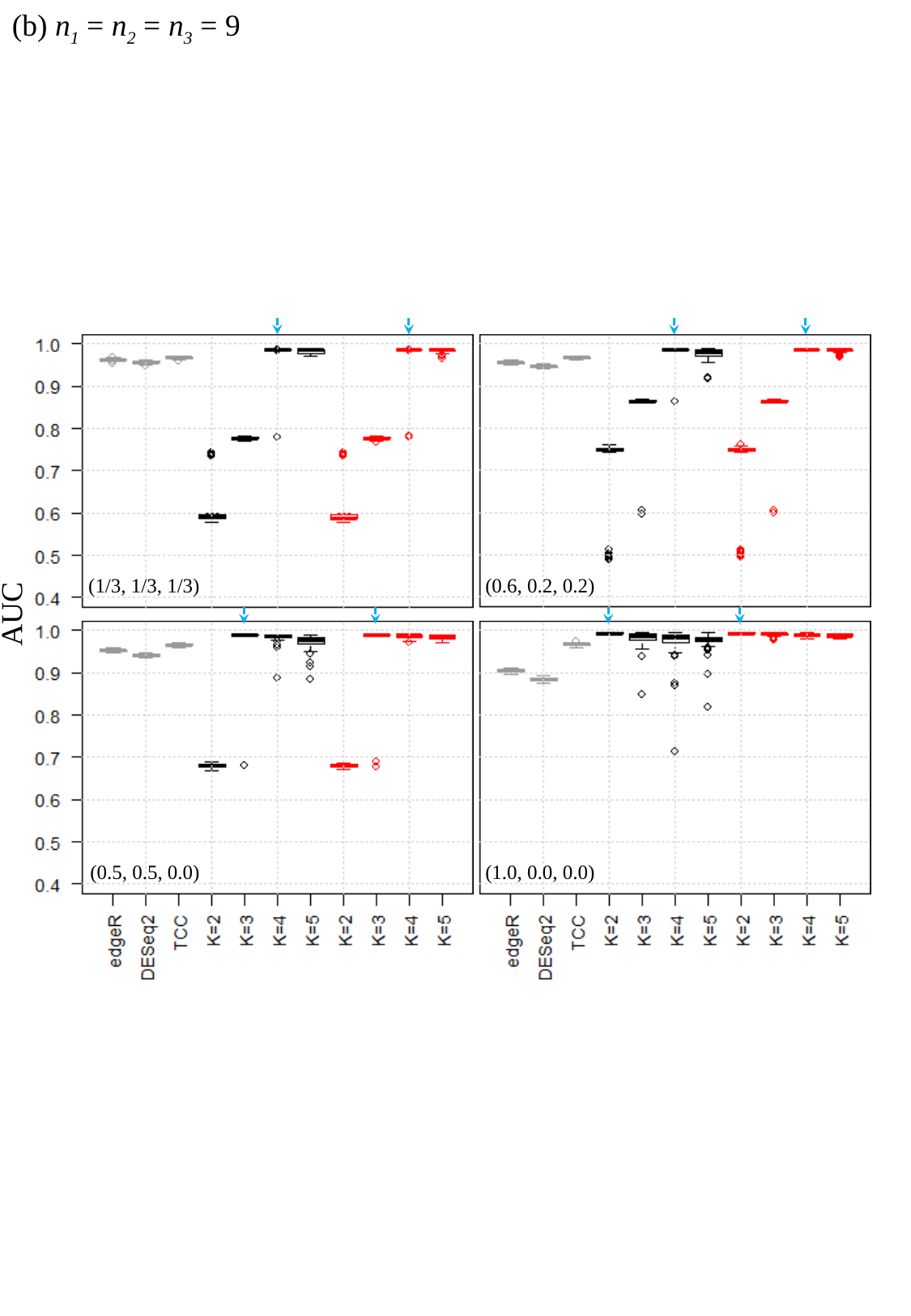

(b) n1 = n2 = n3 = 9
(1/3, 1/3, 1/3)
(0.6, 0.2, 0.2)
AUC
(0.5, 0.5, 0.0)
(1.0, 0.0, 0.0)

## Slide 3
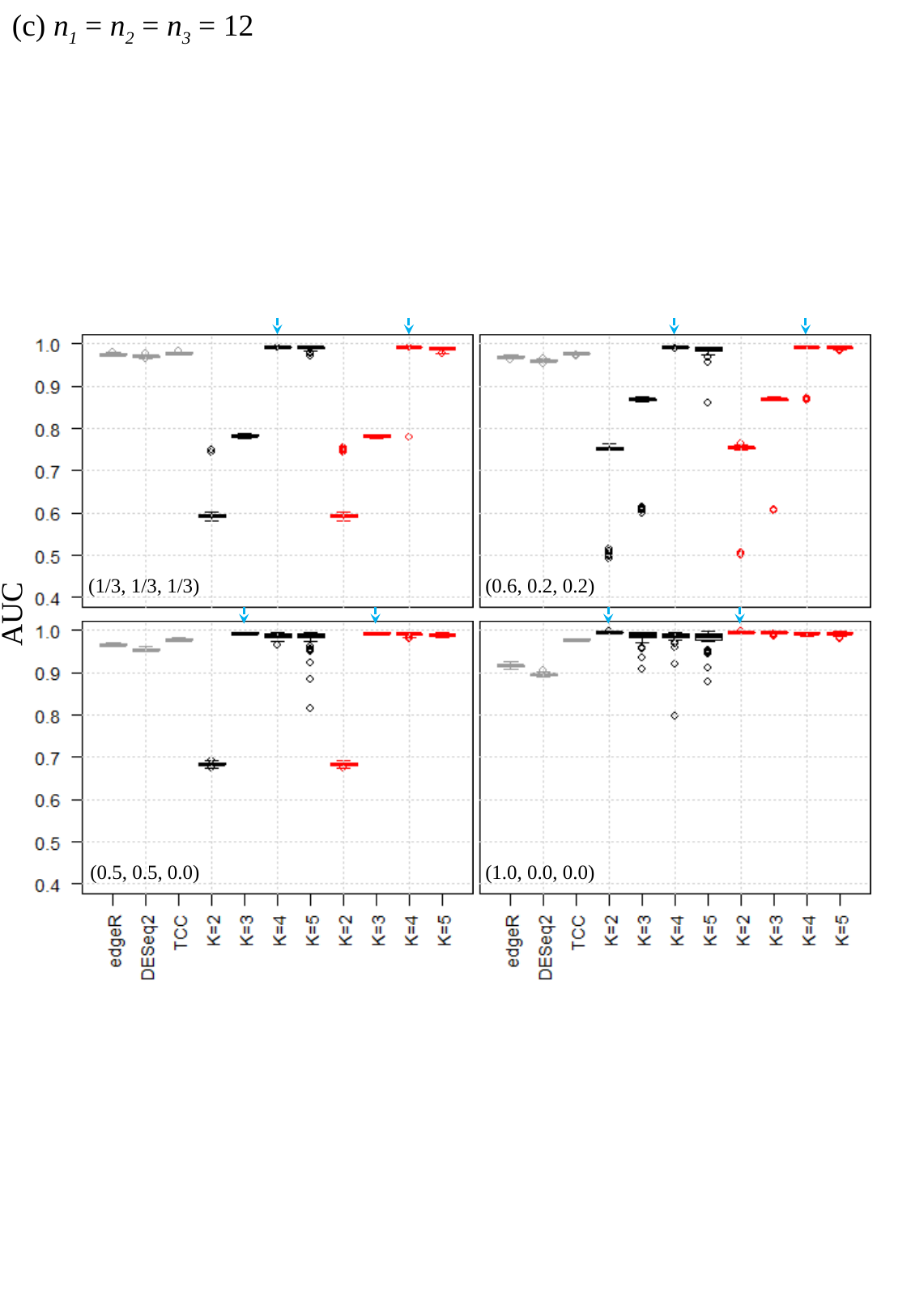

(c) n1 = n2 = n3 = 12
(1/3, 1/3, 1/3)
(0.6, 0.2, 0.2)
AUC
(0.5, 0.5, 0.0)
(1.0, 0.0, 0.0)

Supplement: Supplementary file 6 — Additional file 6. Results corresponding to Fig. 4 with a larger number of replicates. Boxplots of AUC values (50 trials) for individual methods with n1 = n2 = n3 = (a) 6, (b) 9, and (c) 12 are shown. [file 12859_2021_4438_MOESM6_ESM.pptx]

## Slide 1
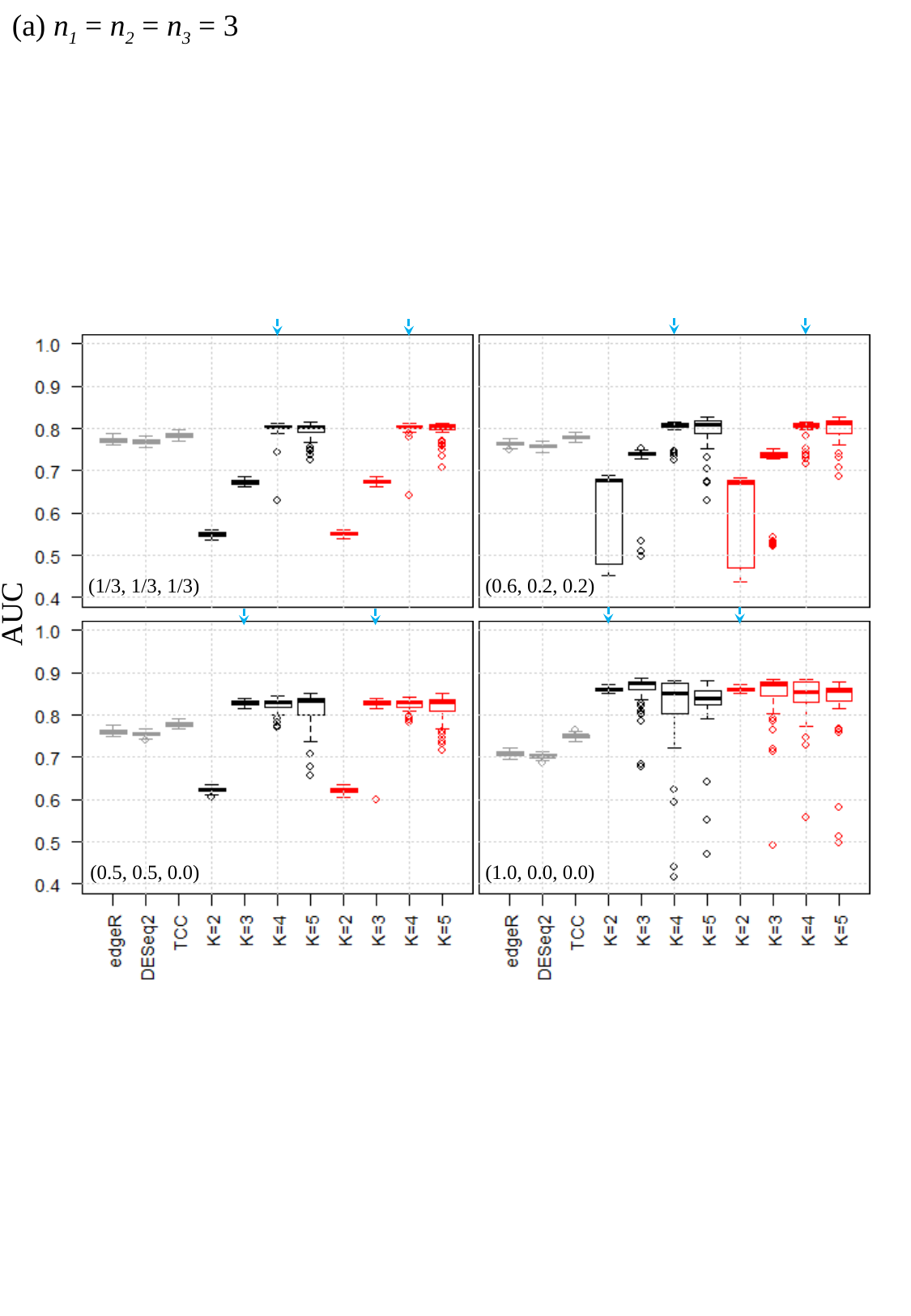

(a) n1 = n2 = n3 = 3
(1/3, 1/3, 1/3)
(0.6, 0.2, 0.2)
AUC
(0.5, 0.5, 0.0)
(1.0, 0.0, 0.0)

## Slide 2
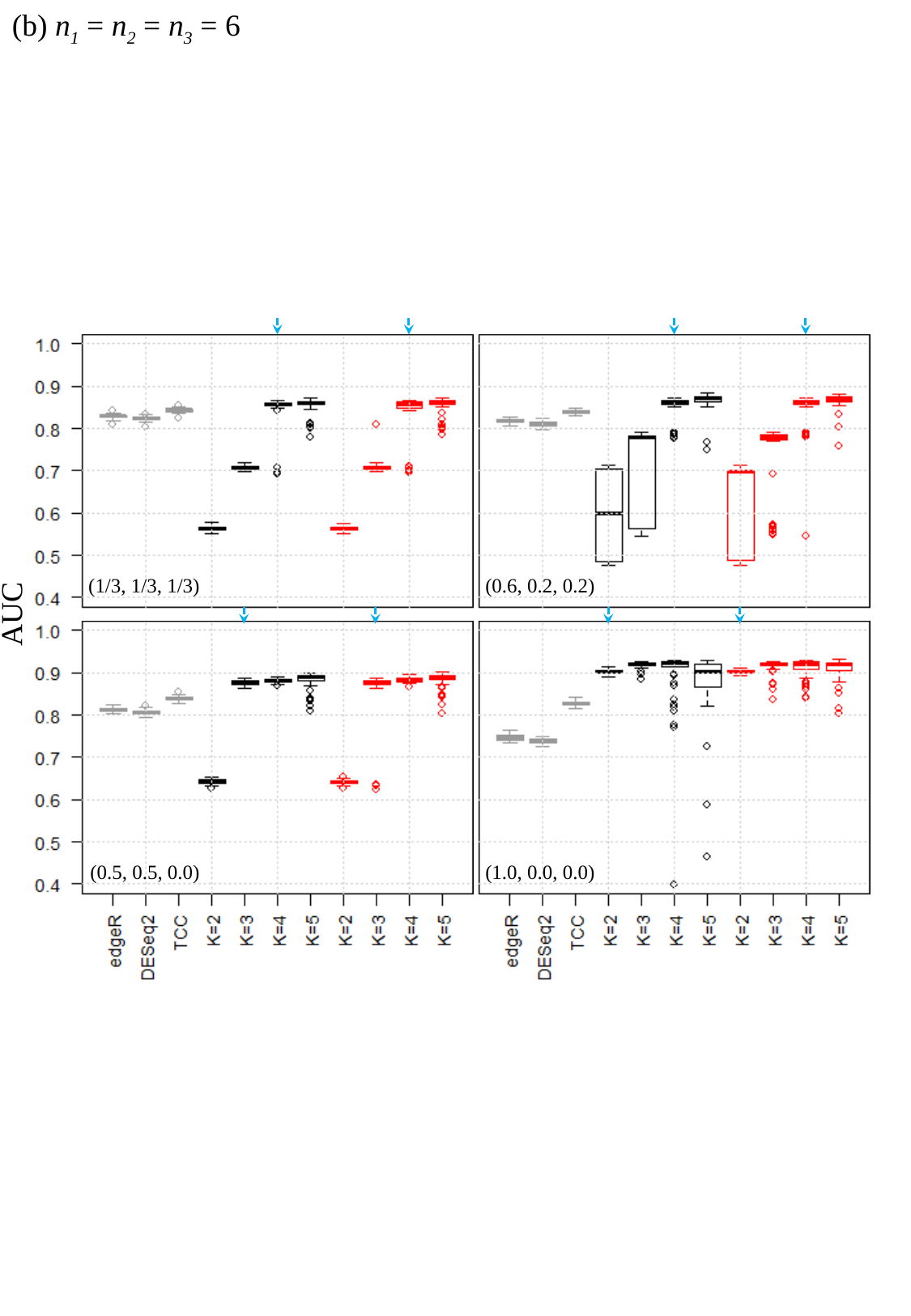

(b) n1 = n2 = n3 = 6
(1/3, 1/3, 1/3)
(0.6, 0.2, 0.2)
AUC
(0.5, 0.5, 0.0)
(1.0, 0.0, 0.0)

## Slide 3
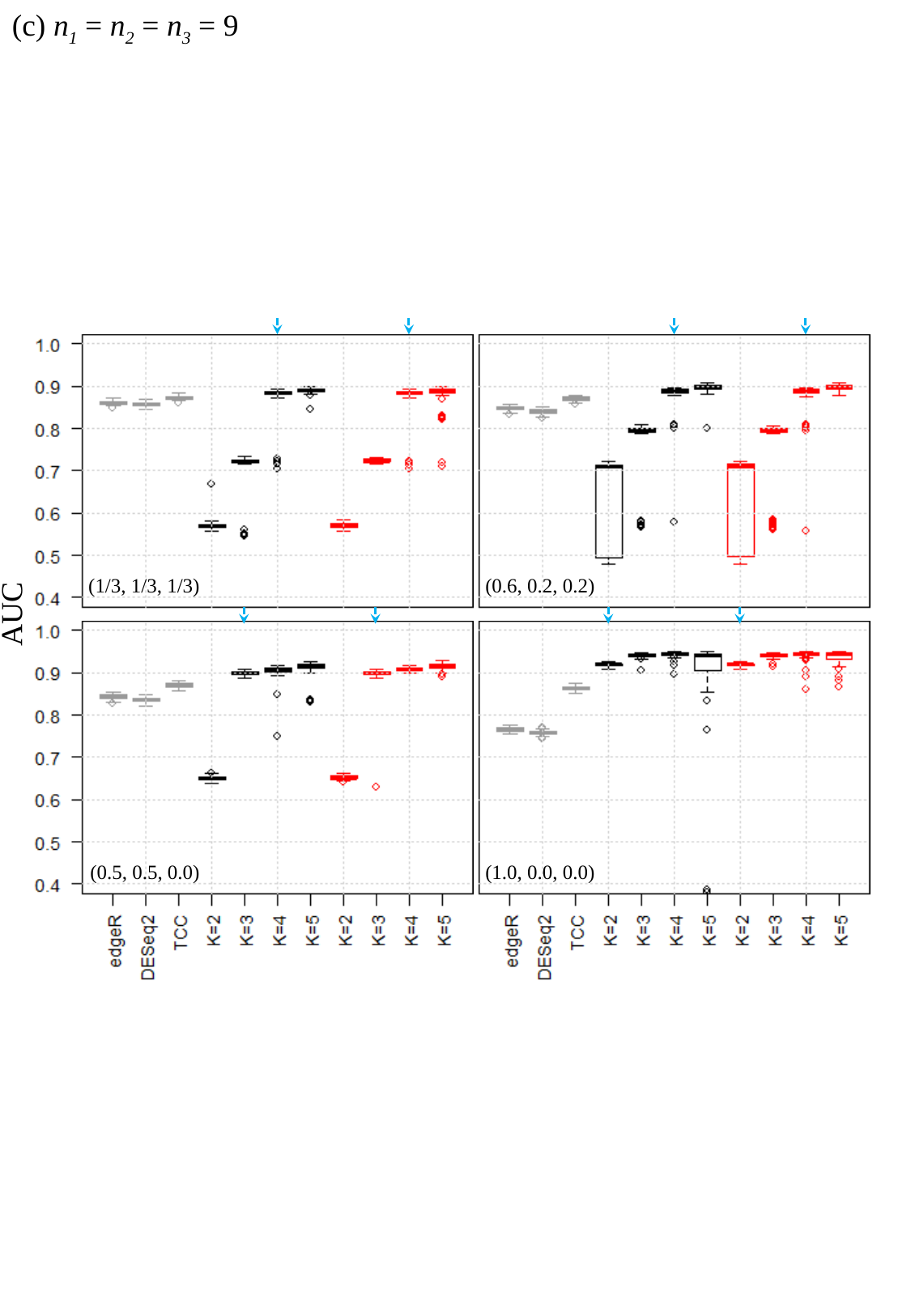

(c) n1 = n2 = n3 = 9
(1/3, 1/3, 1/3)
(0.6, 0.2, 0.2)
AUC
(0.5, 0.5, 0.0)
(1.0, 0.0, 0.0)

## Slide 4
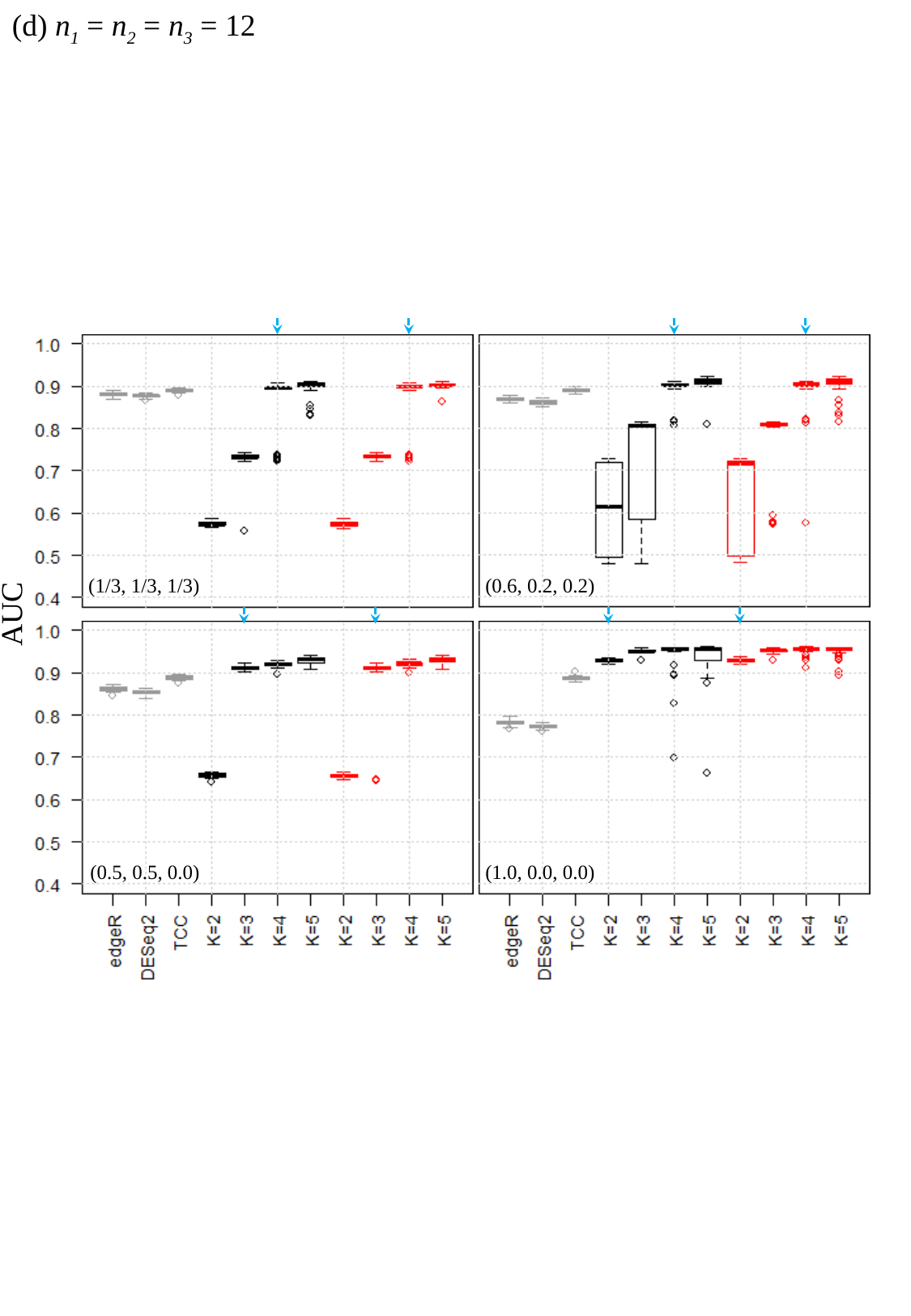

(d) n1 = n2 = n3 = 12
(1/3, 1/3, 1/3)
(0.6, 0.2, 0.2)
AUC
(0.5, 0.5, 0.0)
(1.0, 0.0, 0.0)

Supplement: Supplementary file 7 — Additional file 7. Results corresponding to Additional file 4 on the three-group simulated data. Boxplots of AUC values (50 trials) for individual methods with n1 = n2 = n3 = (a) 3, (b) 6, (c) 9, and (d) 12 are shown. [file 12859_2021_4438_MOESM7_ESM.pptx]
