# Supplementary material for: Differential expression analysis using a model-based gene clustering algorithm for RNA-seq data
Source: BMC Bioinformatics. 2021 Oct 20;22:511. doi: 10.1186/s12859-021-04438-4 (PMC8527798; doi:10.1186/s12859-021-04438-4)
Supplement: Supplementary file 3 — Additional file 3. Effect on different degrees of DE for the five methods. Boxplots of AUC values (100 trials) for individual methods with n1 = n2 = (a) 3, (b) 6, (c) 9, and (d) 12 are shown. In contrast to Fig. 1 and Additional file 1, simulations were performed using different degrees of DE. [file 12859_2021_4438_MOESM3_ESM.pptx]

## Slide 1
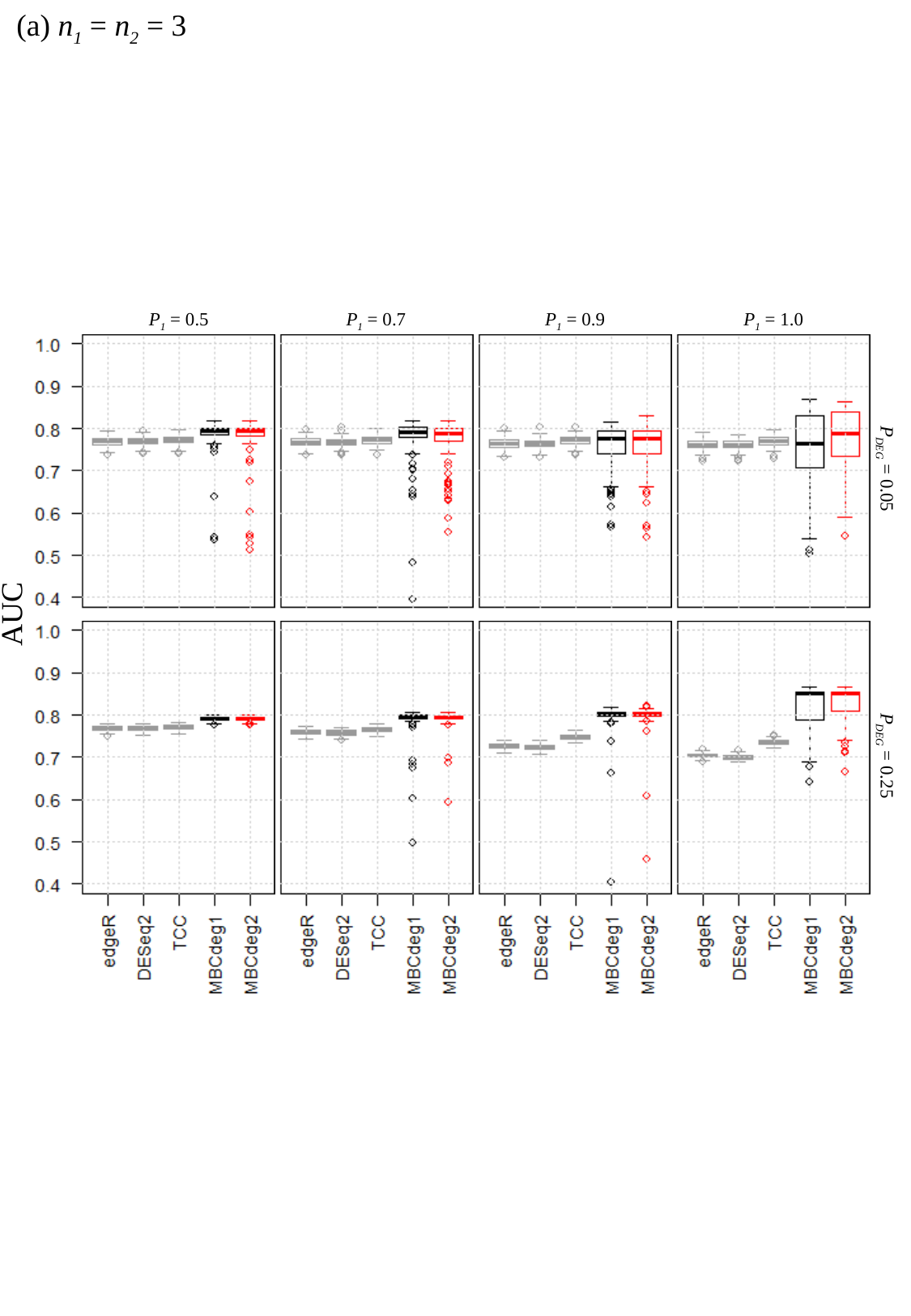

(a) n1 = n2 = 3
P1 = 0.5
P1 = 0.7
P1 = 0.9
P1 = 1.0
PDEG = 0.05
AUC
PDEG = 0.25

## Slide 2
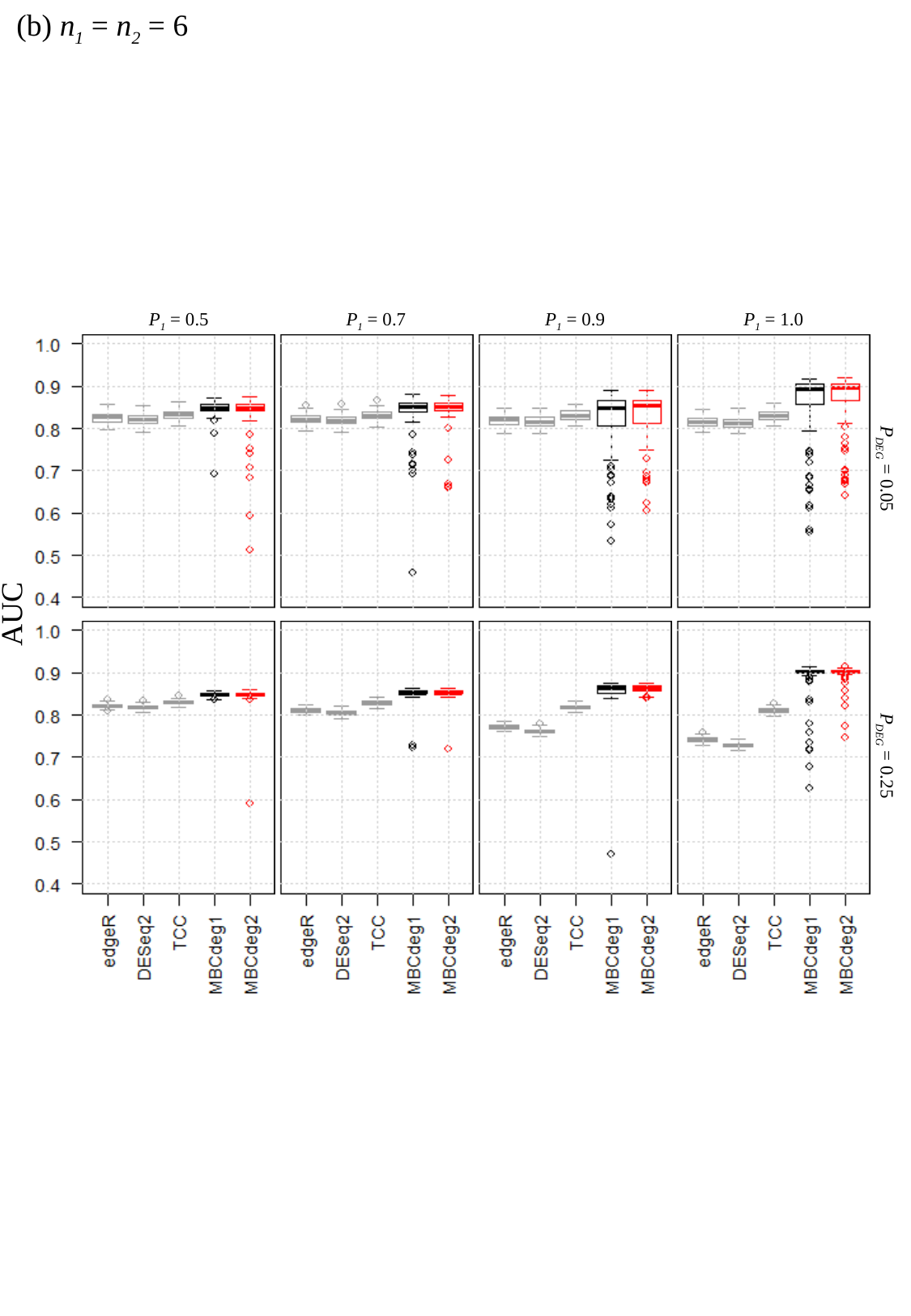

(b) n1 = n2 = 6
P1 = 0.5
P1 = 0.7
P1 = 0.9
P1 = 1.0
PDEG = 0.05
AUC
PDEG = 0.25

## Slide 3
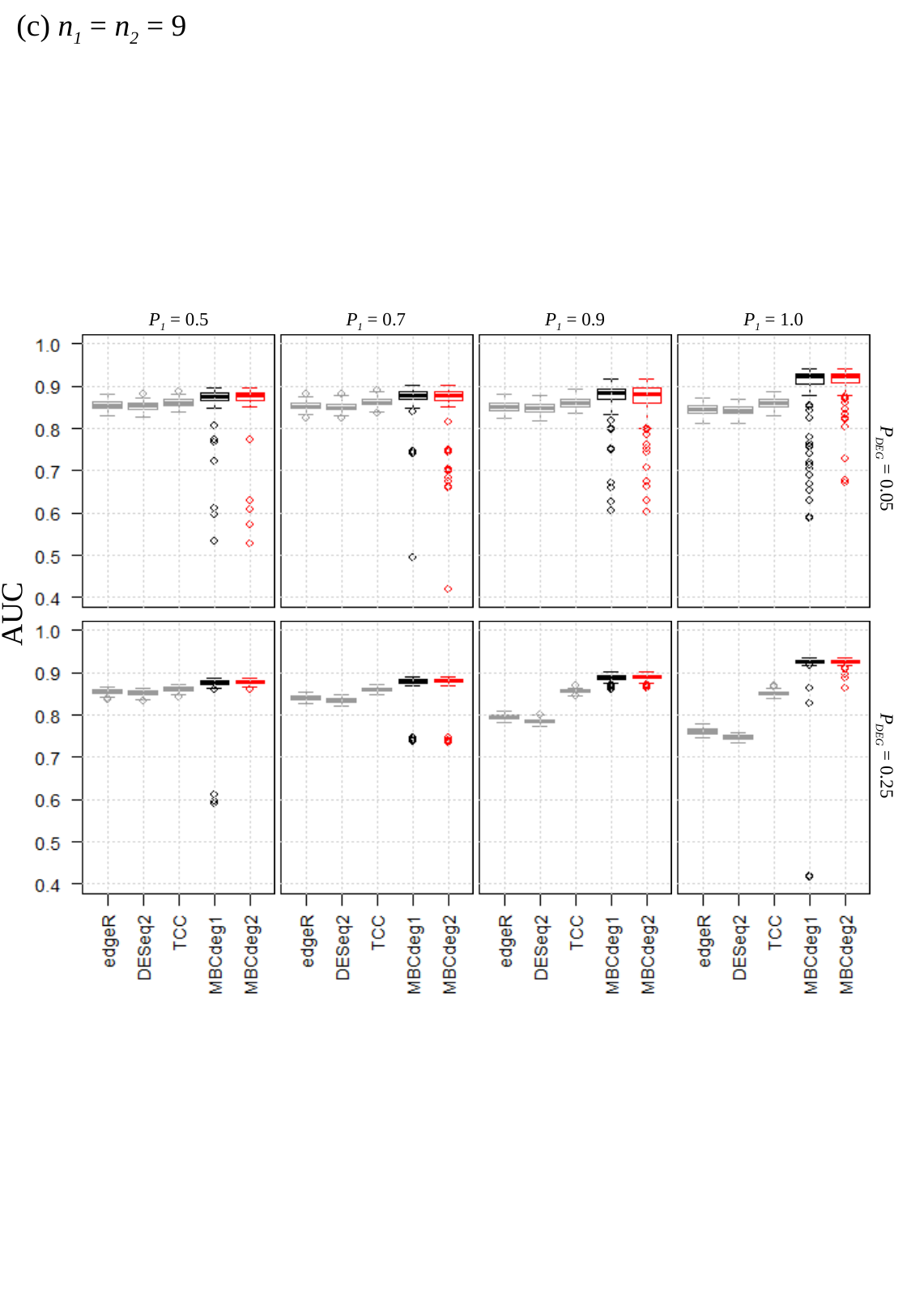

(c) n1 = n2 = 9
P1 = 0.5
P1 = 0.7
P1 = 0.9
P1 = 1.0
PDEG = 0.05
AUC
PDEG = 0.25

## Slide 4
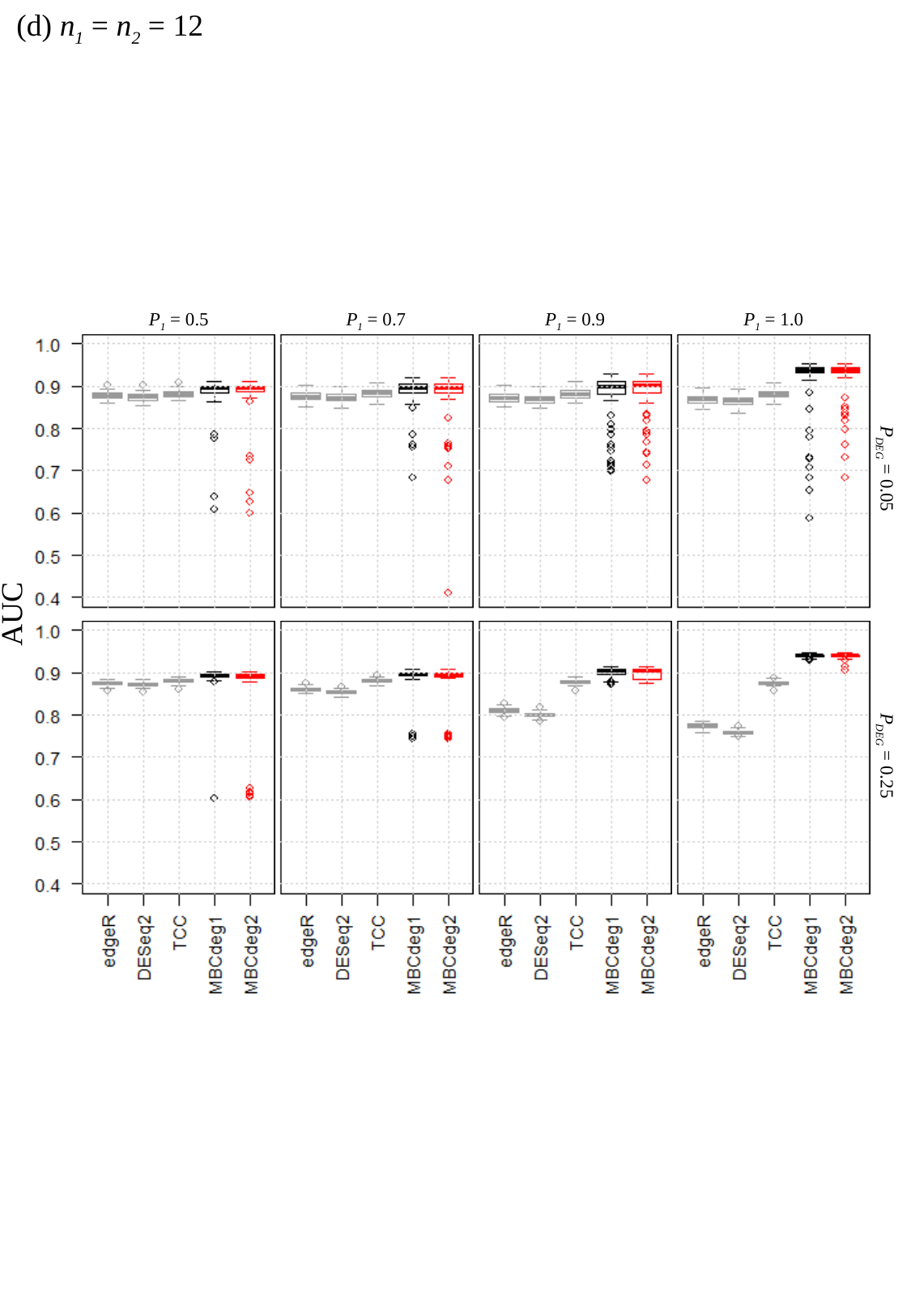

(d) n1 = n2 = 12
P1 = 0.5
P1 = 0.7
P1 = 0.9
P1 = 1.0
PDEG = 0.05
AUC
PDEG = 0.25
