# Supplementary material for: Differential expression analysis using a model-based gene clustering algorithm for RNA-seq data
Source: BMC Bioinformatics. 2021 Oct 20;22:511. doi: 10.1186/s12859-021-04438-4 (PMC8527798; doi:10.1186/s12859-021-04438-4)
Supplement: Supplementary file 4 — Additional file 4. Effect on different degrees of DE for MBCdeg with K = 2–4. Boxplots of AUC values (100 trials) for MBCdeg (K = 2–4) with n1 = n2 = (a) 3, (b) 6, (c) 9, and (d) 12 are shown. In contrast to Fig. 2 and Additional file 2, simulations were performed using different degrees of DE. The AUC values for MBCdeg with K = 3 were almost the same as those in Additional file 3 (different trials were used). [file 12859_2021_4438_MOESM4_ESM.pptx]

## Slide 1
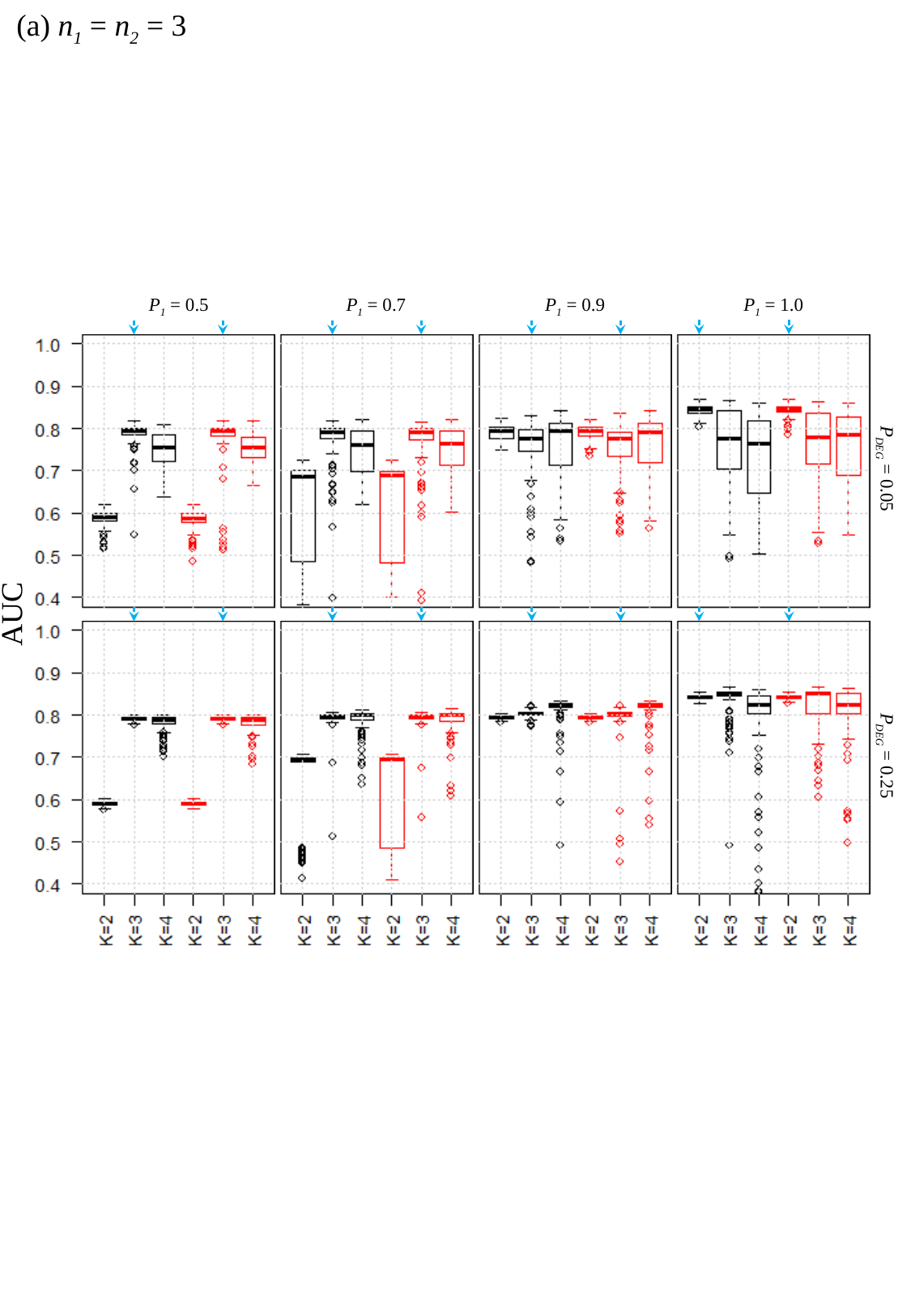

(a) n1 = n2 = 3
P1 = 0.5
P1 = 0.7
P1 = 0.9
P1 = 1.0
PDEG = 0.05
AUC
PDEG = 0.25

## Slide 2
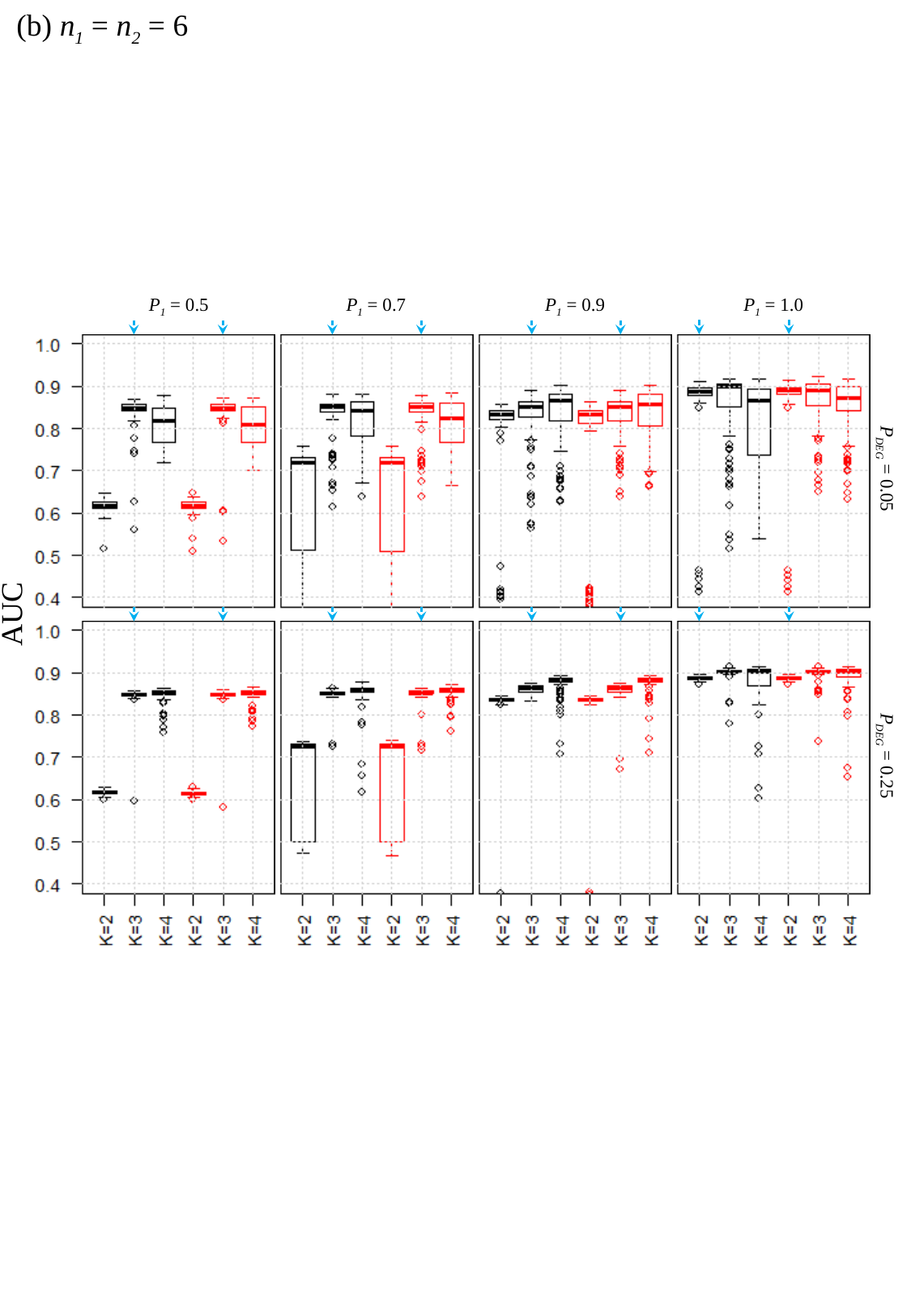

(b) n1 = n2 = 6
P1 = 0.5
P1 = 0.7
P1 = 0.9
P1 = 1.0
PDEG = 0.05
AUC
PDEG = 0.25

## Slide 3
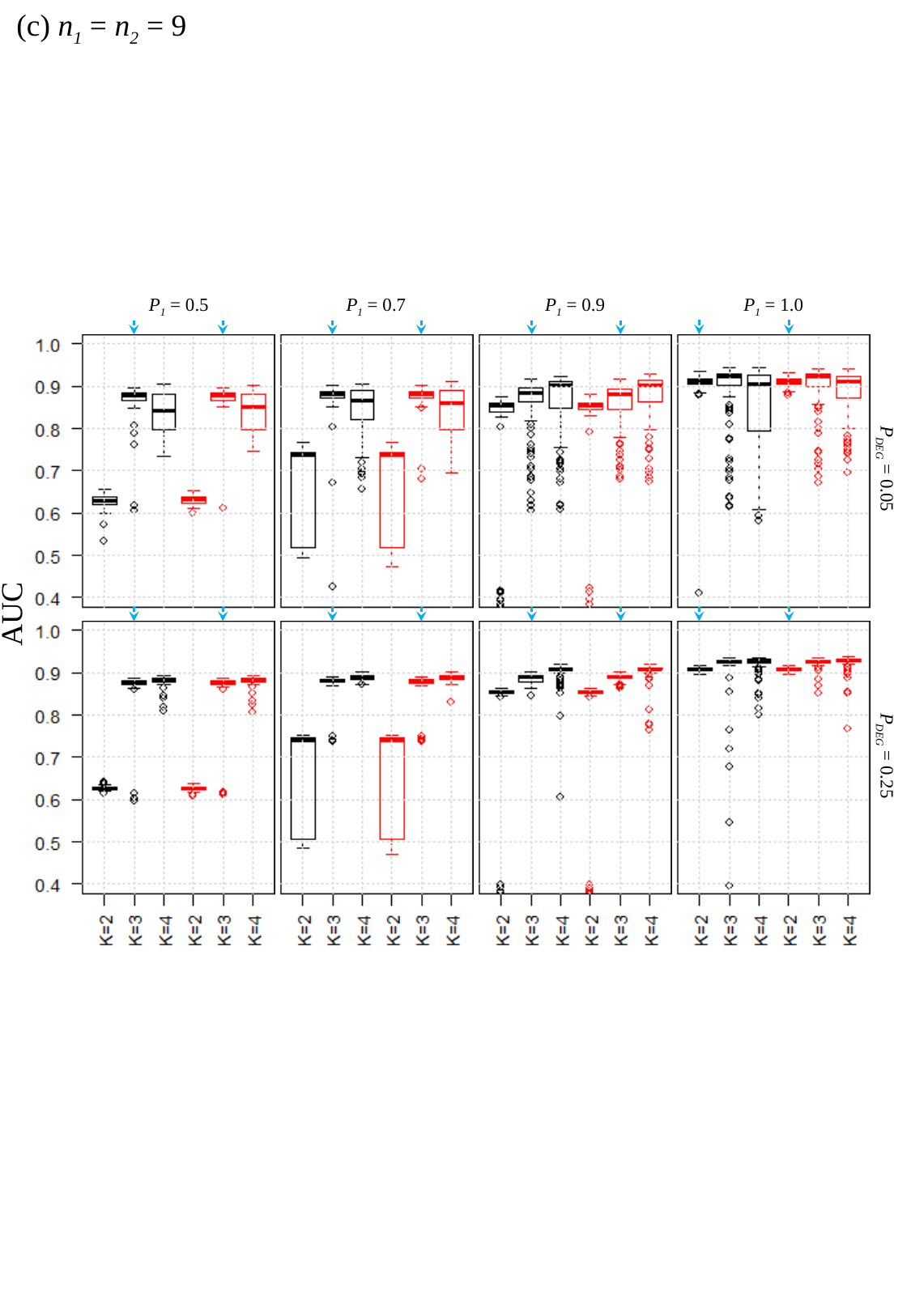

(c) n1 = n2 = 9
P1 = 0.5
P1 = 0.7
P1 = 0.9
P1 = 1.0
PDEG = 0.05
AUC
PDEG = 0.25

## Slide 4
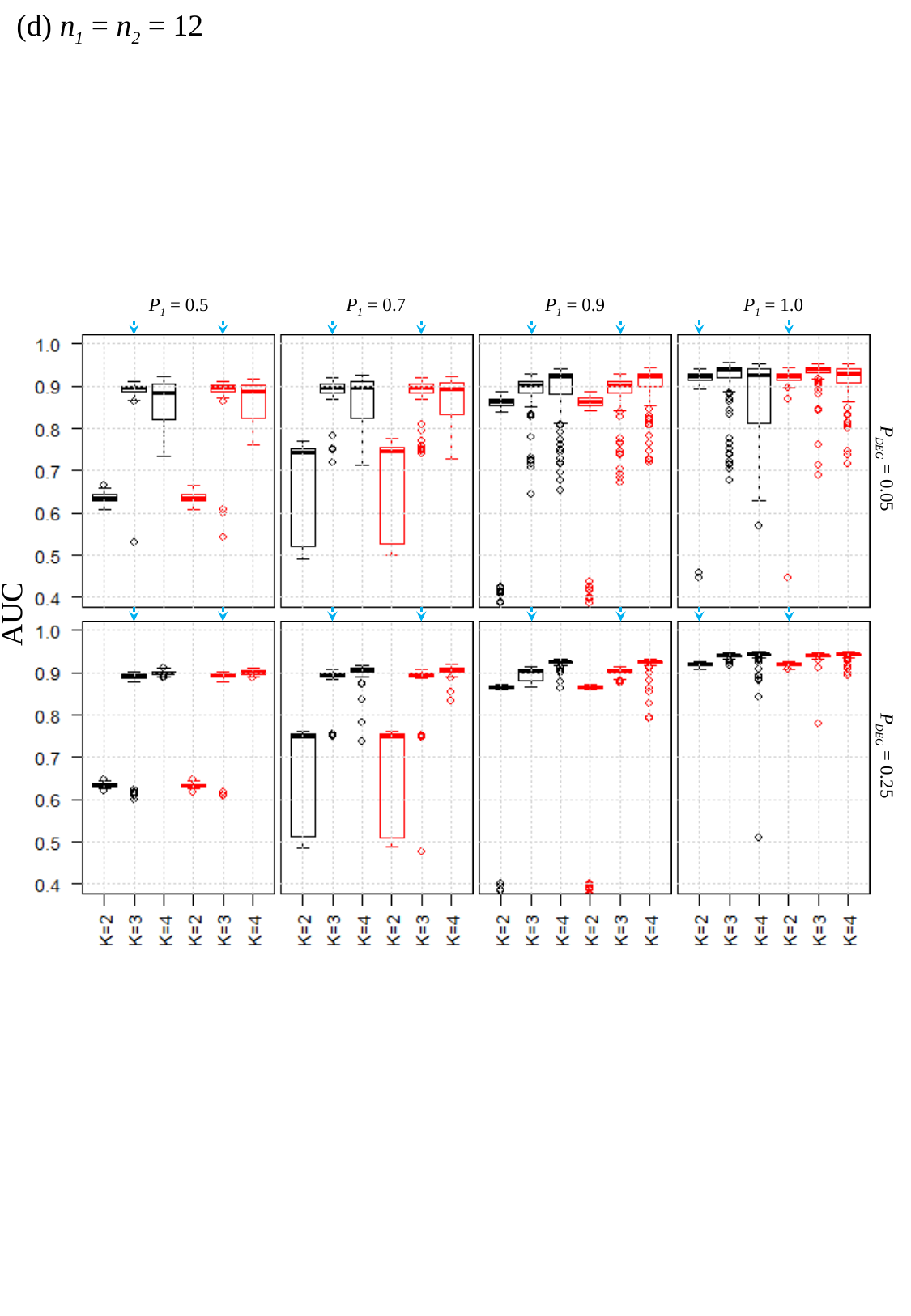

(d) n1 = n2 = 12
P1 = 0.5
P1 = 0.7
P1 = 0.9
P1 = 1.0
PDEG = 0.05
AUC
PDEG = 0.25
