# Supplementary material for: Differential expression analysis using a model-based gene clustering algorithm for RNA-seq data
Source: BMC Bioinformatics. 2021 Oct 20;22:511. doi: 10.1186/s12859-021-04438-4 (PMC8527798; doi:10.1186/s12859-021-04438-4)
Supplement: Supplementary file 5 — Additional file 5. Results corresponding to Fig. 3 with a larger number of replicates. Boxplots of AUC values (50 trials) for individual methods with n1 = n2 = (a) 6, (b) 9, and (c) 12 are shown. [file 12859_2021_4438_MOESM5_ESM.pptx]

## Slide 1
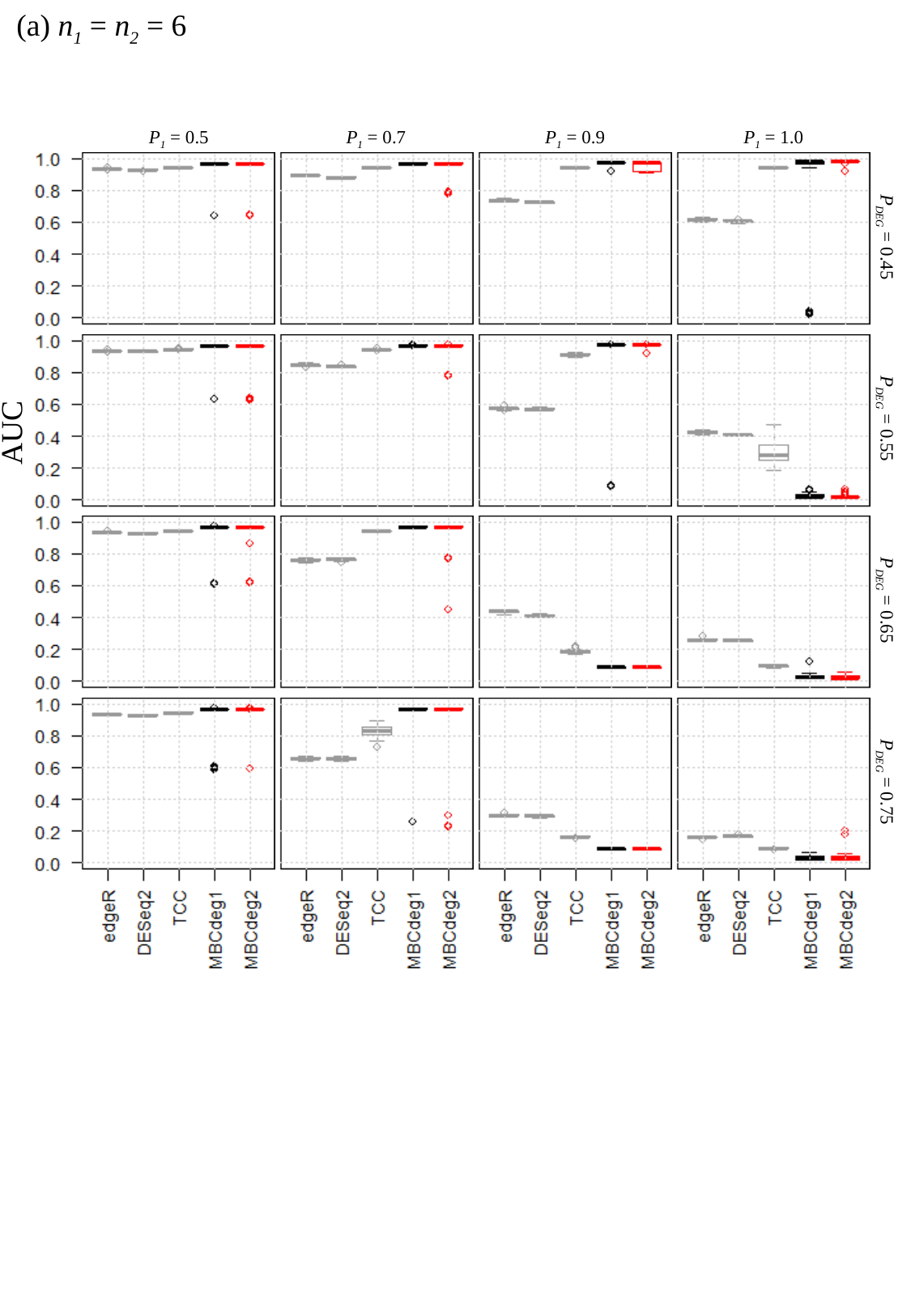

(a) n1 = n2 = 6
P1 = 0.5
P1 = 0.7
P1 = 0.9
P1 = 1.0
PDEG = 0.45
AUC
PDEG = 0.55
PDEG = 0.65
PDEG = 0.75

## Slide 2
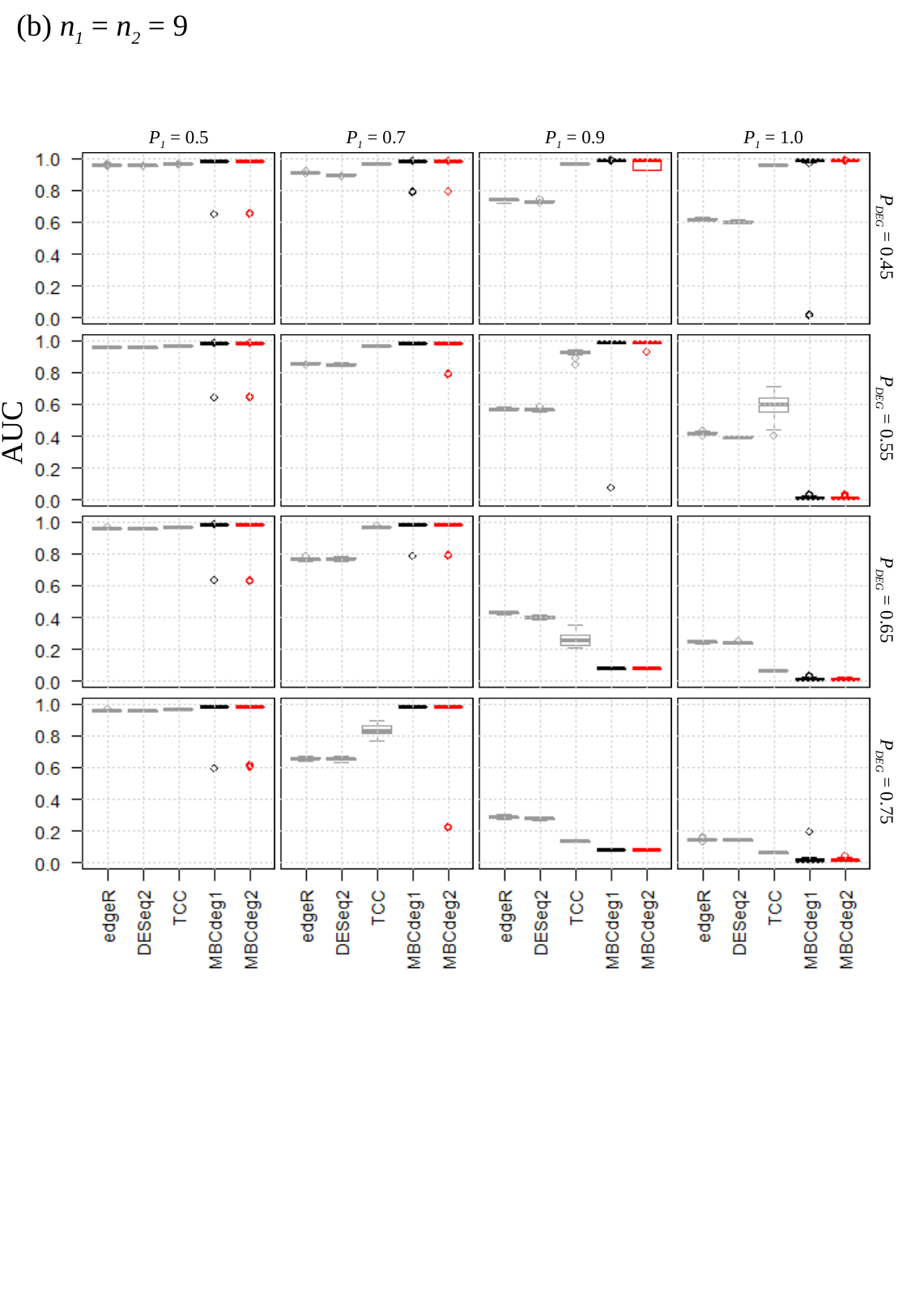

(b) n1 = n2 = 9
P1 = 0.5
P1 = 0.7
P1 = 0.9
P1 = 1.0
PDEG = 0.45
AUC
PDEG = 0.55
PDEG = 0.65
PDEG = 0.75

## Slide 3
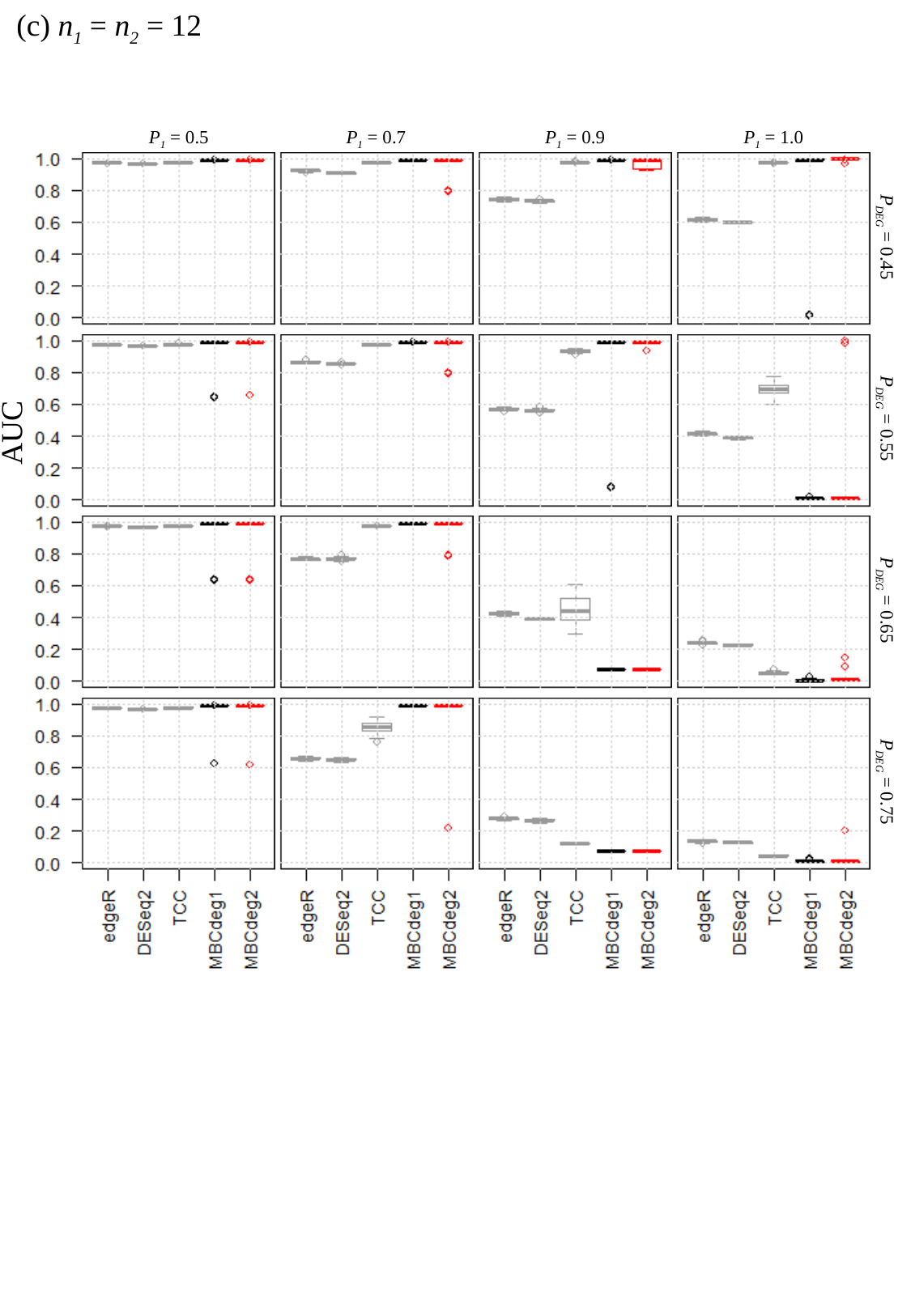

(c) n1 = n2 = 12
P1 = 0.5
P1 = 0.7
P1 = 0.9
P1 = 1.0
PDEG = 0.45
AUC
PDEG = 0.55
PDEG = 0.65
PDEG = 0.75
